# Complex magnetism in a transition metal nitride

**DOI:** 10.1038/s41467-026-78995-8

**Published:** 2026-11-02

**Authors:** Dominik Werhahn, S. Steinberg, Clemens Ritter, Felix Drötboom, J. Paul Attfield, Simon D. Kloß

**Affiliations:** 1 Department of Chemistry, LMU Munich, Munich, Germany; 2 Institute of Inorganic Chemistry, RWTH Aachen University, Aachen, Germany; 3 Institut Laue-Langevin, 71 avenue des Martyrs, CS 20156, Grenoble, Cedex 9 France; 4 Centre for Science at Extreme Conditions, University of Edinburgh, Edinburgh, UK

**Keywords:** Magnetic materials, Solid-state chemistry

## Abstract

High-symmetry lattices of transition metal ions such as the body-centred tetragonal (bct) arrangement are model systems for correlated electron physics and frustrated magnetism. Transition metal oxides or halides with the bct K_2_NiF_4_-type structure are extensively studied and have ground states dominated by strong interactions within the square layers, but materials exemplifying other bct ground states are much rarer. Here we show that Ca_2_CoN_2_, from the underexplored class of transition metal nitrides, has one of the complex magnetic ground states predicted for the bct lattice lying close to a spin-liquid instability. Ca_2_CoN_2_ has been synthesised at high pressure in a large-volume press, and has a Na_2_HgO_2_-type structure where weakening of intralayer superexchange interactions leads to frustration. Our magnetisation and neutron diffraction experiments reveal an incommensurate spin order below 54 K, consistent with a double-*k* spin density wave with propagation vectors (*k*00) and (0*k*0) where *k* = 0.57. This phase lies close to the predicted crossover (at *k* = 0.64–0.69) to a modulated spin liquid, which could be explored through further chemical tuning. This work demonstrates the potential of transition metal nitrides as magnetic model systems, alongside the much-studied oxides and halides, for exploring complex spin orders and associated quantum phases.

## Abstract (web summary)

Model magnets bordering spin-liquid instabilities are usually transition metal oxides or halides. Here, the authors show that the nitride Ca2CoN2 orders as an incommensurate double-k spin density wave close to a predicted spin-liquid phase, establishing nitrides as magnetic model systems.

## Introduction

Studies of body-centred tetragonal (bct) lattices of transition metal spins are dominated by K_2_NiF_4_-type (*n* = 1 member of the A_*n*+1_B_*n*_*X*_3*n*+1_ Ruddlesden-Popper layered perovskite family) materials based on oxide or halide anions. Mott insulator Sr_2_IrO_4_, unconventional superconductor Sr_2_RuO_4_, high-T_c_ superconducting cuprates based on La_2_CuO_4_, and spin and charge ordered analogues based on La_2_NiO_4_ are prominent model compounds that have led to new understanding of exotic ground states, phase transitions, and exchange interactions^1–3^. These properties are governed by strong B–X–B magnetic superexchange ( ~ 100 meV) between nearest neighbours in square BX_2_ layers^2,4^, while next-neighbour interactions ( ~ 1–10 meV) are much weaker^5,6^. Interlayer couplings, however, determine 3-dimensional magnetic orders, for example, competing pseudodipolar and interlayer exchange interactions control spin canting in Sr_2_IrO_4_, while in La_2_CuO_4_ weak interlayer coupling stabilises three-dimensional order, and in La_2_NiO_4_ weak anisotropies drive spin reorientation at structural transitions^7–9^. These examples highlight the importance of weaker exchange interactions on the bct lattice, which are, however, often shrouded by the strong B–X–B superexchange.

These nearest-neighbour magnetic interactions in K_2_NiF_4_-type materials are greatly diminished by removing the anions within the BX_2_ square layers, giving the Na_2_HgO_2_-type structure with unusual linear 2-coordination of B-site transition metals. The resulting long metal-metal interatomic distances lead to weak nearest-neighbour (d_J2–J2_ > 3.5 Å) and next- nearest-neighbour metal-metal exchange interactions (d_J3–J3_ > 5.0 Å). Examples for this structure type are families of uranium and thorium carbides U_2_*M*C_2_ (*M* = Ir, Pt, Os, Ru, Rh) and Th_2_NiC_2_, oxonickelates *A*_2_NiO_2_ (*A* = K, Rb, Cs), and nitridometallates *AE*_2_ZnN_2_ (AE = Ca, Sr, Ba) and Ca_2_NiN_2_^10–16^. The carbides are itinerant electron systems; U_2_RhC_2_ and U_2_IrC_2_ have AFM transitions at T_N_ = 22 K and 7 K, respectively, while Th_2_NiC_2_, U_2_PtC_2_ and the solid solutions U_2_Pt_x_Rh_1−x_C_2_ are superconductors below 8.5 and 1.5 K, respectively^10,17,18^. The only reported low-temperature magnetic measurements for the oxo- and nitridonickelates with localised spins have revealed apparent short-range AFM orders with broad maxima at T_N_ = 20 K for Cs_2_NiO_2_^13^, and at ca. 200 K for Ca_2_NiN_2_^16^. Overall, few Na_2_HgO_2_-types with localised spins are known and experimental determinations of ordered magnetic structures have not been reported.

Transition metal nitrides with itinerant electrons have interesting magnetic ground states, such as in the permanent magnet Fe_16_N_2_ or Mn-based nitrides for non-collinear antiferromagnetic spintronics^19,20^. Nitrides with complex magnetic structures of localised moments, however, remain scarce, but are worthwhile to study owing to the polar-covalent *M*–N bonding that should strengthen superexchange interactions, as demonstrated for nitride spinel MnTa_2_N_4_^21^. Therefore, preparation of magnetic anion-defect ordered Ruddlesden-Popper nitrides is of interest for studying the weaker interactions governed by superexchange. Access to Na_2_HgO_2_-type *AE*_2_MN_2_ nitridometallates has been limited by the difficulty of stabilising the transition metal in a M^+II^ state, which is relatively high in nitrides of late transition metals. A high-pressure and high-temperature route using a large-volume press and an active nitriding agent (azide) has recently been used to prepare highly oxidised nitrides such as nitride perovskites, K_2_NiF_4_-type nitrides and nitridometallates such as Ca_4_Fe^+IV^N_4_ and Na_2_HgO_2_-type Ca_2_NiN_2_^16,22–25^. Here we report Ca_2_CoN_2_ with Co in the +II oxidation state that is unprecedented for nitridocobaltates. Magnetic neutron diffraction reveals a double-k spin density wave producing a checkerboard-type magnetic structure in proximity to a predicted spin liquid phase. This complex magnetic structure evidences frustration in the bct lattice, which we propose arises from long Co–N–Ca–N–Co superexchange pathways^26^.

## Results

### Preparation and structure determination

**Fig. 1:**
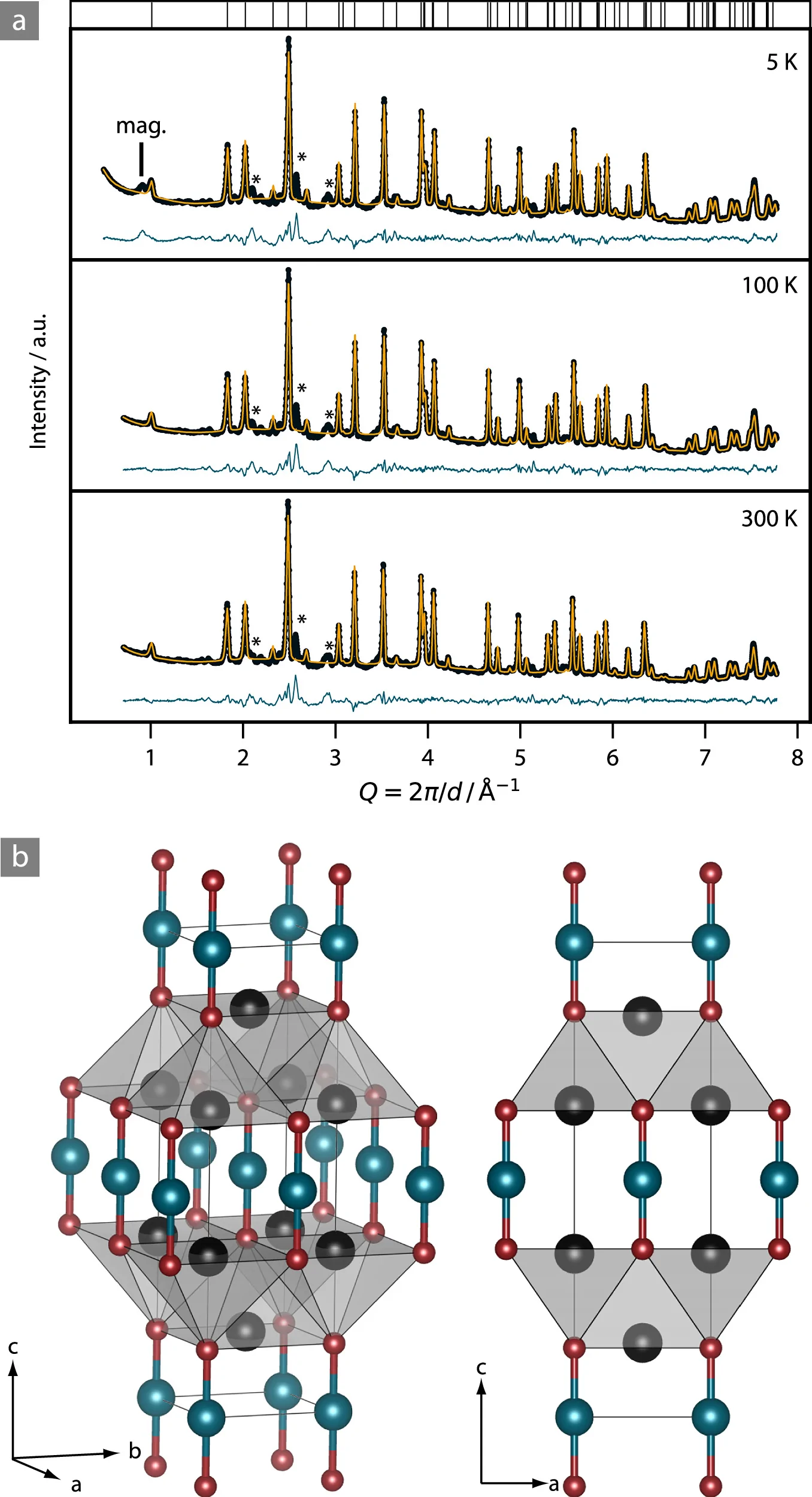
Powder neutron diffraction data and crystal structure of Ca_2_CoN_2_. **a** Rietveld refinement of PND data of Ca_2_CoN_2_ collected at 300, 100 and 5 K at the D20 beamline of the ILL. Data as black circles, Rietveld fit as orange line, difference curve as blue line, calculated nuclear Bragg peak positions marked as vertical lines. Unknown impurity reflections marked with asterisks and a prominent magnetic reflection marked in the 5 K measurement. **b** Two views of the structure of Ca_2_CoN_2_ in the Na_2_HgO_2_-type, with N in red, Co in blue and Ca in black with grey CaN_5_ polyhedra.

Ca_2_CoN_2_ was prepared through reaction of Ca_3_N_2_, Co metal, and NaN_3_ at 8 GPa and 800 °C in a multianvil large-volume press. A 22.5 wt-% excess of Ca_3_N_2_ was required to suppress the formation of unidentified byproducts (Eq. 1). The samples were obtained as air-sensitive black powders that contained small amounts of an unknown impurity (ca. 5% by estimation of relative peak intensities), which probably contains the excess Ca/Na/N (Fig. 1a).

1 $$2+{{{\rm{ex\,C}}}}{{{{\rm{a}}}}}_{3}{{{{\rm{N}}}}}_{2}+3\,{{{\rm{Co}}}}+2\,{{{\rm{Na}}}}{{{{\rm{N}}}}}_{3}\to {3\,{{{\rm{Ca}}}}}_{2}{{{{\rm{CoN}}}}}_{2}+2\,{{{\rm{Na}}}}+2\,{{{{\rm{N}}}}}_{2}$$

Powder diffraction data of Ca_2_CoN_2_ were obtained on in-house X-ray diffractometers (Supplementary Fig. S1) as well as at the neutron D20 high-intensity two-axis diffractometer (Fig. 1a) of the Institut Laue-Langevin (ILL). Discussion of the structure presented here is based on the neutron data, details of all collected data are listed in Supplementary Tables S1–S4. Tentative refinement of nitrogen occupancies on neutron data indicates full nitridation and a bulk composition of Ca_2_CoN_2_. All atomic positions were freely refined, and anisotropic displacement parameters could be refined for all three atomic sites for the powder X-ray diffraction data, while for powder neutron diffraction (PND) only N and Ca could be refined anisotropically.

Ca_2_CoN_2_ crystallises in the tetragonal space group *I*4/*mmm* (no. 139) in the Na_2_HgO_2_-type (Fig. 1b) with lattice parameters *a*_295K_ = 3.57175(9), *c*_295K_ = 12.4127(6) Å and *V*_295K_ = 158.35(1) Å^3^ (Details in Supplementary Information). The structure is composed of layers of [Co^II^N_2_]^4−^ dumbbells, separated by layers of square-pyramidal CaN_5_, where Ca resides on the face of the N_4_-square basal plane. The Co-atoms are arranged in a square lattice with an in-plane interatomic distance equal to the *a/b* lattice parameters (*a* = 3.57 Å), a next-nearest-neighbour interatomic distance (*ab*-plane diagonal) of 5.05 Å, while the inter-layer distance equals half the c-axis (*c*/2 = 6.2 Å). The structure is related to previously reported Ca_5_Co^I^_2_N_4_, which can be seen as a defect-ordered variant of the Na_2_HgO_2_-type with vacancies of CoN_2_ dumbbell moieties^27^. The Co–N bond lengths are 1.796(7) Å, which is similar to Li*AE*_2_[CoN_2_] (*AE* = Ca, Sr) *d*_Co–N_ = 1.79–1.81 Å, and shorter than in Ca_5_Co^I^_2_N_4_ (d = 1.821(4) Å) and the Na_2_HgO_2_-type materials *A*_2_ZnN_2_ (*A* = Ca, Sr, Ba) with *d*_Zn–N_ = 1.84–1.87 Å, and longer than in Ca_2_NiN_2_ with *d*_Ni–N_ = 1.748(3) Å^14–16,27,28^. Ca–N interatomic distances *d*_Ca–N_ = 2.488(5) and 2.5300(3) Å are comparable to other reported distances in Ca_4_FeN_4_, Ca_2_NiN_2_, and Ca_2_ZnN_2_ (*d*_Ca–N_ = 2.41–2.54 Å)^14,16^.

The thermal stability of Ca_2_CoN_2_ was investigated with temperature dependent powder X-ray diffraction (Supplementary Fig. S2) in the region between 50 and 950 °C. Up to 300 °C, both lattice parameters *a* and *c* increase, while at higher temperatures, a structural change is suggested by a decreasing *c* lattice parameter. Decomposition with formation of new reflections sets in at around 600 °C. The structural changes may be related to partial reduction of Co, as has been observed for Ca_2_NiN_2_, which decomposes to CaNiN. The observed reflection patterns, however, do not match those of CaCoN or Ca_5_Co_2_N_4_. Linear expansion coefficients were fitted for *a* (60 to 500 °C), *c* (60 to 300 °C), and *V* (60 to 300 °C), resulting in α_a_ = 1.78(3)·10^−5^ K^−1^, α_c_ = 1.7(2)·10^−5^ K^−1^, and α_V_ = 5.6(3)·10^−5^ K^−1^, which is similar to values found in Ca_2_NiN_2_ (α_a_ = 1.8(1)·10^−5^ K^−1^ and α_c_ = 7.1(6) 10^−6^ K^−1^ ^)16^.

### Magnetic properties

**Fig. 2:**
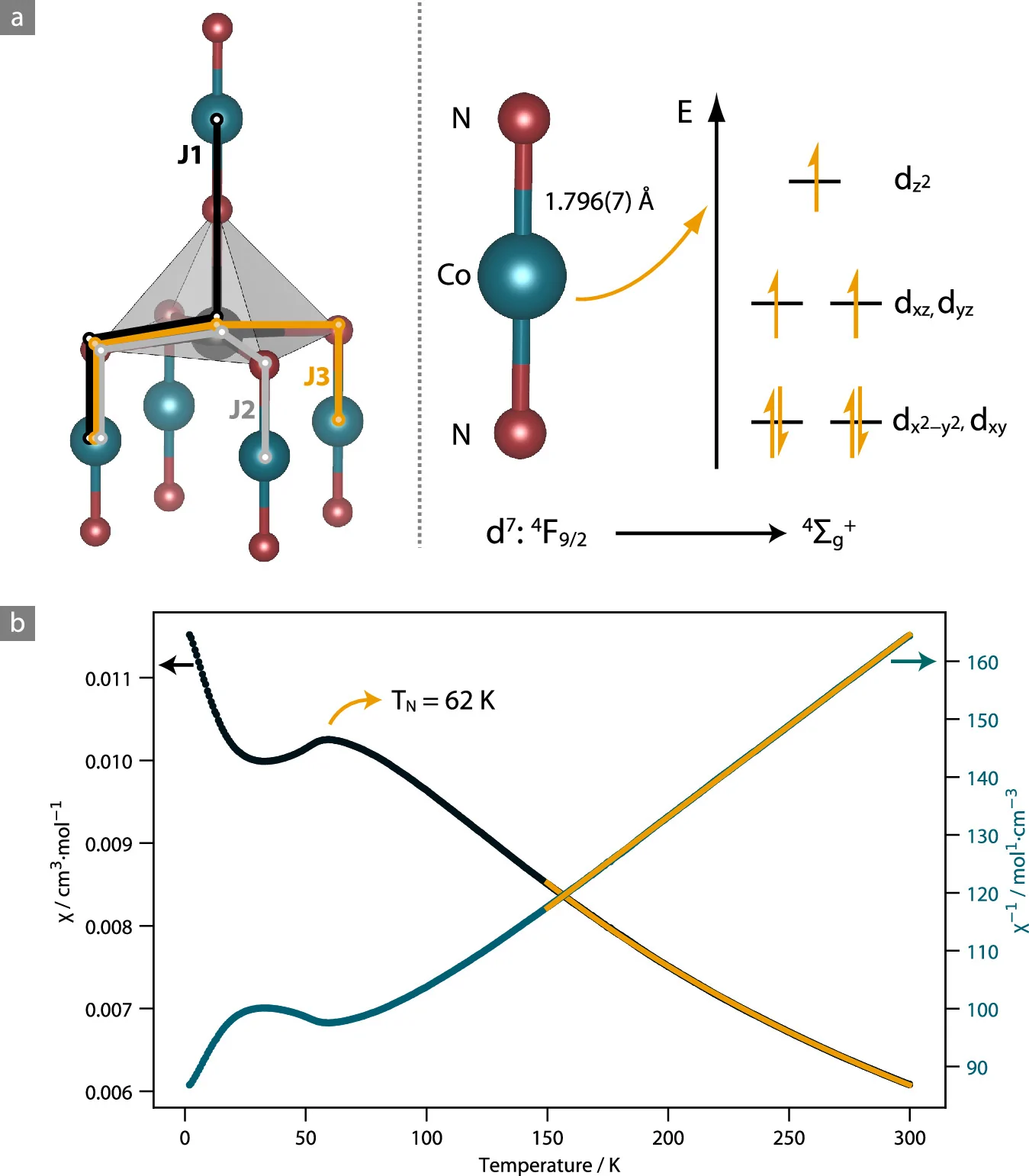
Exchange pathways, d-orbital splitting, and susceptibility measurements of Ca_2_CoN_2_. **a** (Left) Possible exchange pathways Co–N–Ca–N–Co in Ca_2_CoN_2_. *J*_2_ indicates nearest-neighbour, *J*_3_ next-nearest-neighbour, and *J*_1_ interplane interactions. (Right) Linear [CoN_2_]^4−^ dumbbell with crystal field splitting pattern shown on the right. The crystal field splits the ^4^F_9/2_ ground state of the free Co^2+^ ion into several components, with a non-degenerate ^4^Σ_g_^+^ ground state^30^. **b** Susceptibility data of Ca_2_CoN_2_ obtained in a field of 30 kOe. Black circles are recorded *χ*_M_, blue reciprocal *χ*_M_^−1^, orange is a Curie-Weiss fit.

The magnetic susceptibility of Ca_2_CoN_2_ was investigated in a field of 30 kOe in the range of 2–300 K (Fig. 2). At high-temperature the susceptibility follows the Curie-Weiss model, and a fit (details in SI) in the region from 150 to 300 K yielded a magnetic moment of *µ*_eff_ = 5.04(1) μ_B_ (mass correction applied for the surplus of Ca_3_N_2_ used in the reaction) and a Weiss temperature Θ = − 222.7(2) K, in line with localised d-electron magnetism. For linear D_∞h_ coordination, the crystal field splitting of the Co^II^ d^7^ system produces a ^*4*^Σ_g_ ground state from an (d_x2–y2_, d_xy_)^4^(d_xz_,d_yz_)^2^(d_z2_)^1^ high-spin electron configuration with quenched orbital momentum and an effective moment of *µ*_eff_ = 3.87 µ_B_ (Fig. 2) that is lower than the observed value. First order spin-orbit coupling would require a non-Aufbau configuration for Co^+II^ as observed for very weak ligands, and is not likely here^29^. For stronger ligands such as nitride anions, the orbital momentum is introduced by mixing the ground state with low-lying excited states^30–32^. Following this mechanism, linear Co^2+^ complexes with L–M–L angles of 180° often show enlarged effective magnetic moments such as Co[N(SiMe_3_)Dipp]_2_ (*µ*_eff_ = 4.9 µ_B_) and Co[N(H)Ar^Pri6^]_2_ (*µ*_eff_ = 6.3 µ_B_)^30,33^.

The large negative Weiss temperature reflects antiferromagnetic exchange interactions, which are manifested in a broad Néel-type transition with a maximum susceptibility at *T*_max_ = 62 K. This is similar to the isostructural Ca_2_NiN_2_ where the square-planar arrangement of Ni^2+^ with S = 1 led to low-dimensional magnetism with a broad maximum in the susceptibility at 200 K, although long-range spin ordering was not demonstrated^16^. In Ca_2_CoN_2_, the broadened transition and a frustration parameter *f* = |Θ|/T_N_ = 3.6 indicate significant magnetic frustration. Such frustration in a bct lattice originates from multiple exchange pathways (Fig. 2a). The nearest-neighbour Co−Co distances within the square lattices (3.57 Å) are too long for direct exchange interactions, as suggested for Cs_2_NiO_2_^13^. Therefore, longer superexchange pathways Co–N–Ca–N–Co *J*_2_ (nearest-neighbour, 90°), *J*_3_ (next-nearest-neighbour, 180°), and *J*_1_ (interplane, 90°) are likely to transmit spin information in accordance with previous analyses of bct lattices^34^. The bonding and possible exchange interactions are further investigated in the density functional theory section.

### Magnetic neutron diffraction

**Fig. 3:**
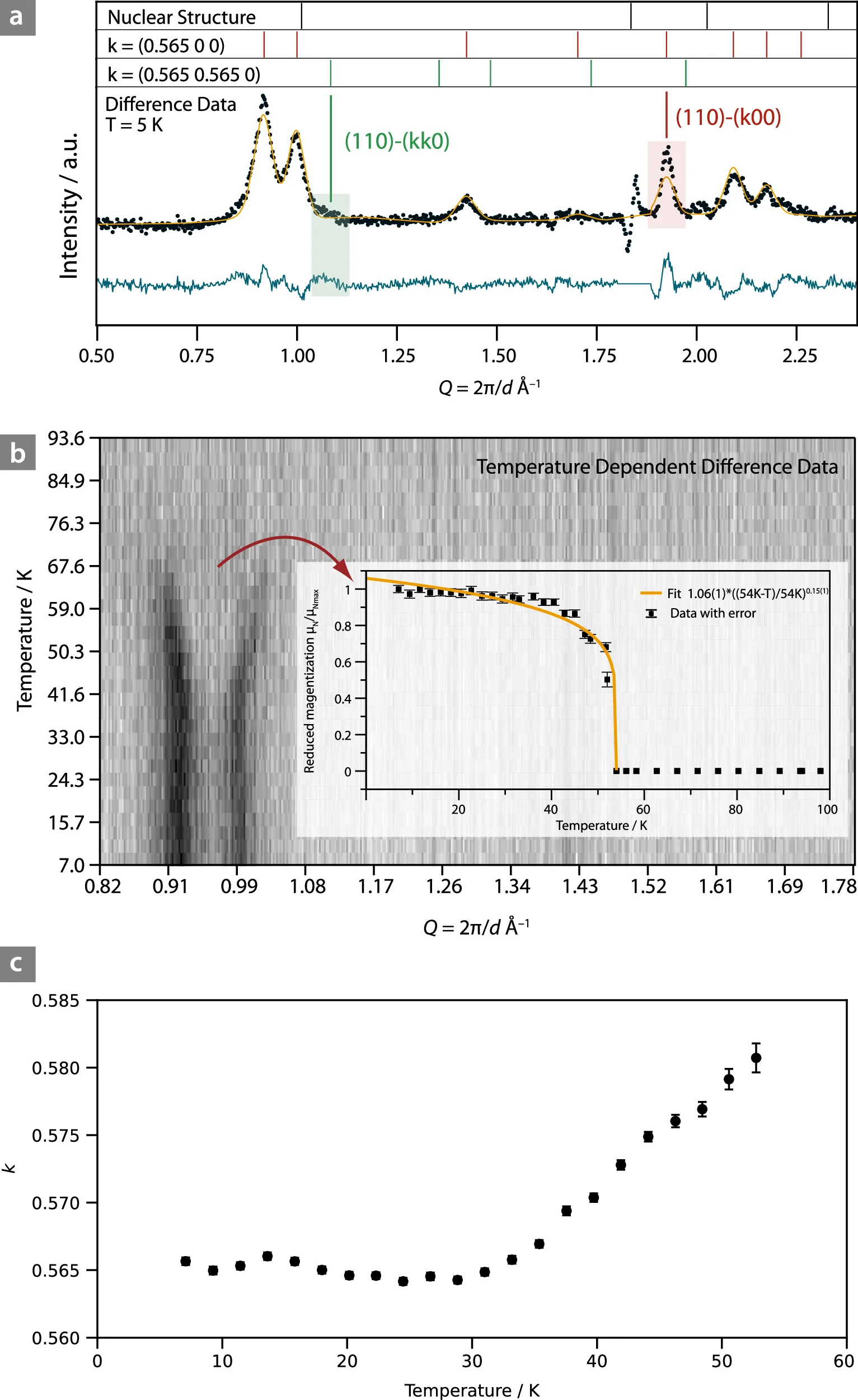
Magnetic powder neutron diffraction data of Ca_2_CoN_2_. **a** Fit of the incommensurate magnetic structure to difference neutron diffraction data *I*_*5K*_-*I*_100K_ with data in black, fit in orange, difference below in blue. Markers at the top show expected positions of nuclear structure reflections (black), first-order *k*_1_ = (0.565 0 0) magnetic structure reflections, and second-order *k*_1_ + *k*_2_ = (0.57 0.57 0) reflections. The weak second-order (110) − (0.57 0.57 0) reflection is highlighted. (Excess intensity is also observed at the low-*Q* side of the magnetic peaks at *Q* = 0.91 Å^−1^, but this results from peak asymmetry, which does not generate extra intensity at the high-*Q* side). **b** Temperature-dependent magnetic neutron diffraction data displayed as difference *I* − *I*_94K_. Inset shows a mean field fit to the obtained magnetic moments with *T*_N_ = 54 K. **c** Temperature-variation of the *k*-vector refined from the temperature-dependent data showing a lock in transition at around 30 K. For (**b**, **c**), data points were obtained from Rietveld refinement of *n* = 22 independent temperature-dependent neutron diffraction patterns, each representing a single measurement at the stated temperature; error bars represent one standard deviation as estimated from the least-squares refinement.

To further investigate the magnetic structure, powder neutron diffraction data were collected on the high-intensity powder diffractometer D20 (Institut Laue-Langevin, Grenoble, France, details in the experiment section of the Supplementary Information): long scans at 5, 46, and 100 K as well as temperature-dependent diffraction from 7 to 98 K (Fig. 3 and Supplementary Fig. S8) were collected. Magnetic reflections are visible in the difference data (*I*_*5K*_–*I*_100K_ and *I*_46K_–*I*_100K_), and these were indexed by an incommensurate magnetic propagation vector *k*_1_ = (0.5665(1) 0 0) (from long scan at 5 K) using the programme K-Search, which is part of the Fullprof suite of programmes^35^. As the vectors *k*_1_ = (0.5665(1) 0 0) and *k*_2_ = (0 0.5665(1) 0) are equivalent in powder diffraction measurements, the possibilities of a spin structure with either single-*k*, double-*k* with two magnetic single-*k* domains, or double-*k* within one domain cannot be directly distinguished without further single-crystal measurements.

However, a single-*k* structure (Space Group *Immm*.1’(0,0,g)s00s^36,37^, as presented in Supplementary Information, Supplementary Fig. S4) entails ferromagnetic alignment of spins in one axis direction within the *ab*-plane, while in the other spins are oriented antiparallel. This appears physically unlikely given that exchange pathways in the *a* and *b* directions are equivalent (Fig. 2a), furthermore such different magnetic couplings along *a* and *b* directions would likely distort the lattice to lower (orthorhombic) symmetry, but no peak splittings or broadenings are observed in the low temperature neutron data. As the neutron data have poor Δ*Q*/*Q* resolution and are insensitive toward changes in lattice parameters, additional synchrotron diffraction data were collected at the ID22 high-resolution powder diffractometer beamline of the ESRF facility, at temperatures of 100 K and 4 K, above and below the transition^38^. These also indicate that the tetragonal metric is retained through the magnetic phase transition (see Supplementary Information for further information). Moreover, some evidence is given for the double-k structure by observation of a higher-order magnetic reflection from the combined *k*_1_ + *k*_2 _= (*kk*0) = (0.57 0.57 0) propagation vector. A strong first order (110)−(*k*00)/ (110)−(0*k*0) magnetic peak is observed at *Q* = 1.92 Å^−1^, and the corresponding second order (110)−(*kk*0) reflection is observed as a weak intensity at a d-spacing of 5.79 Å (*Q* = 1.08 Å^−1^) in the 5 K − 100 K difference data (Fig. 3a). No further higher harmonic reflections that might indicate squaring-up of the spin structure at low temperatures are observed. Hence, a double-*k* structure is taken to be the most likely possibility as a uniform magnetic ground state that is compatible with tetragonal symmetry.

**Fig. 4:**
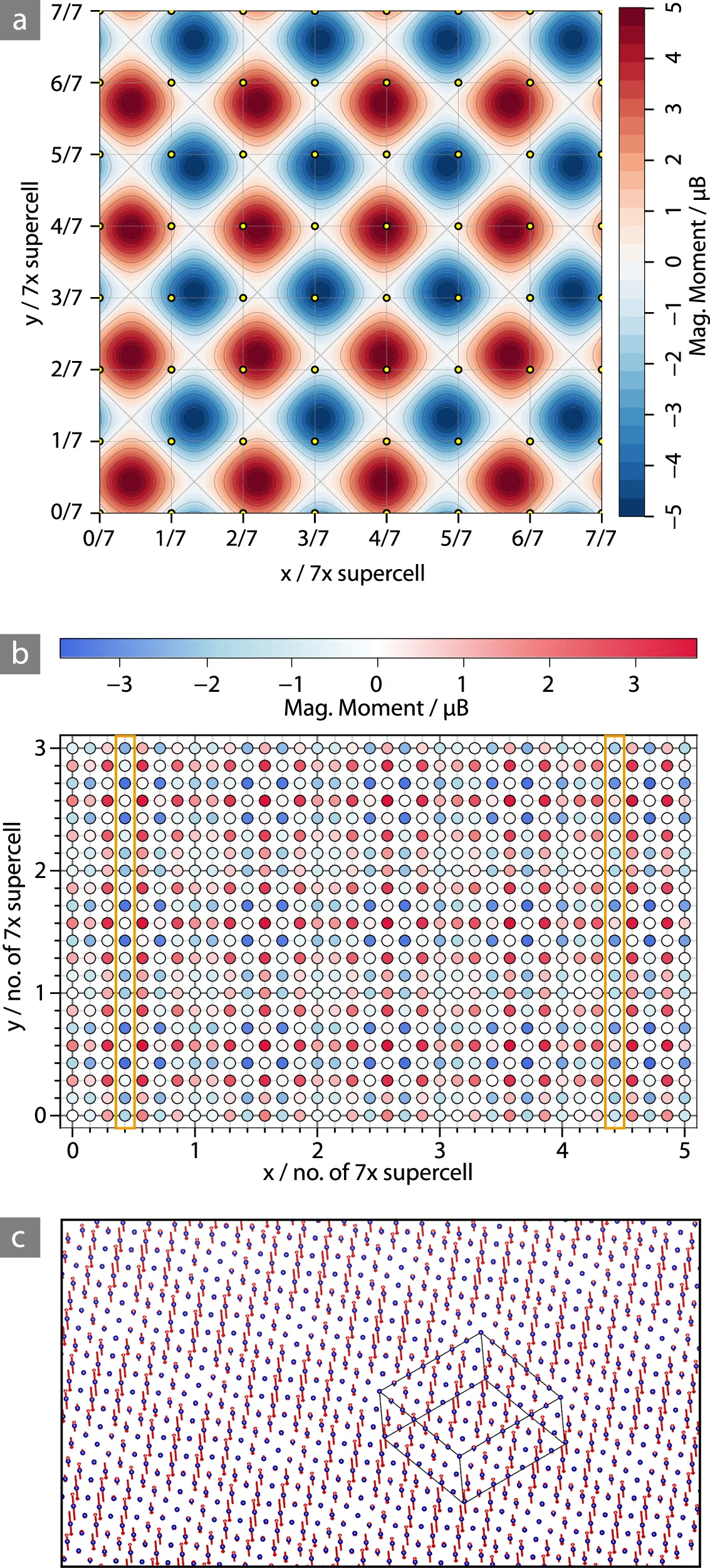
Magnetic double-*k* structure of Ca_2_CoN_2_. **a** Contour plot of the interference pattern created by the double-k spin density waves. Shown is a representative 7×7 superstructure, where individual cobalt atom positions (yellow circles) are located at the intersections. Positive magnetisation direction in red, negative in blue. The plot highlights the local magnetic structure with next to zero magnetic moment on nearest neighbours and antiferromagnetic alignment on next nearest neighbours. **b** Long-range magnetic structure projected on the *ab*-plane in the region of a 3 by 5 array of a 7 × 7 superstructure. Magnetic moment directions are indicated by red/blue colour. Orange rectangles highlight the magnetisation propagation owing to the incommensurate modulation. Regions of high and low total magnetisation are discernible by colour intensity. **c** Magnetic structure plotted as a commensurate approximation calculated with a 7 × 7 superstructure. Regions of high and low total magnetisation are discernible by vector lengths.

The double-*k* magnetic structure (Space Group *I*4/*mmm*.1’(*a*,0,0)0000 *s*(0,*a*,0)00*s*0*s*)^36,37^ can be described as two interfering amplitude modulated spin density waves running in orthogonal directions *a* and *b* (Fig. 4a). The magnetic moment modulation is a superposition of sine waves $\mu \left({{{\rm{x}}}},{{{\rm{y}}}}\right)=\,{\mu }_{\max }{{{\rm{\cdot }}}}\left[\sin \left(2{{{\rm{\pi }}}}{{{\rm{\cdot }}}}\frac{k{{{\rm{\cdot }}}}x}{b}\right)+\sin \left(2{{{\rm{\pi }}}}{{{\rm{\cdot }}}}\frac{k{{{\rm{\cdot }}}}y}{b}\right)\right]\,=\,{\mu }_{\max }{{{\rm{\cdot }}}}\left[\sin \left(2{{{\rm{\pi }}}}{{{\rm{\cdot }}}}q{{{\rm{\cdot }}}}{n}_{x}\right)+\sin \left(2{{{\rm{\pi }}}}{{{\rm{\cdot }}}}q{{{\rm{\cdot }}}}{n}_{y}\right)\right]$, with *x*,*y* being the position of the Co atoms and *n*_x,y_ = the number of the unit cells along *a* and *b*. The maximum amplitudes were determined with *μ*_max_ (5 K) = 5.00(3) µ_B_ and *μ*_max_(46 K) = 3.21(7) µ_B_. As the observed *k* = 0.57 ≈ 4/7, a 7 × 7 superstructure (Fig. 4c) can serve as a commensurate approximation. The magnetic moments are oriented strictly parallel and antiparallel to the c-axis, which reflects orbital contribution that creates strong easy-axis anisotropy in the Co^2+^ ions. The sine-wave interference pattern creates a checkerboard of spin up and down moments, leading on a local scale to non-magnetic nearest neighbours and antiferromagnetically aligned next-nearest neighbours (Fig. 4a). As the dimensions of the checkerboard are, however, incommensurate with the structural lattice, the long-range resulting magnetic structure manifests as larger-scale regions of high and low magnetisation. The high and low magnetisation regions form a square net with lattice spacing of about *λ*_SN_ = 4 *λ*_5K_ = 2.5 nm (Fig. 4b, c). The scale of the pattern, despite the strictly uniform magnetisation direction along *c*, is reminiscent of ultra-small size magnetic skyrmion lattices as found in Gd_3_Ru_4_Al_12_, where the Ruderman−Kittel−Kasuya−Yosida (RKKY) interaction leads to helical pitches with 2.8 nm size^39,40^. The Co atoms in the layers above and below have a phase-shift of *x* + 0.5 and *y* + 0.5, resulting in regions of high magnetisation in one layer coinciding with a region of low magnetisation of the adjacent layers. Introducing phase shifts for both sine waves retains the long-range structure and only shift the complete structure in *a* and *b* direction, respectively.

The maximum observed magnetic moment at low temperatures is larger than the expected value from only spin contributions (*gS* = 3 µ_B_), which is in line with the observed non-quenched orbital momentum (*gJ* = 6 µ_B_ for the free Co^2+^ ion). Similar unquenched orbital contributions were reported for CoO (*µ* = 4 µ_B_) as well as oxide sulfides *AE*_2_CoO_2_Cu_2_S_2_ (*AE* = Sr, Ba) with *µ*_Sr_ = 3.8 µ_B_ and *µ*_Ba_ = 4.5 µ_B_^41,42^. The maximum saturation in Ca_2_CoN_2_, moreover, is reached only in regions of high total magnetisation, the mean magnetisation is around 40.6 % due to destructive interference of the spin-density waves with *µ*_mean_ = 2.0 µ_B_. The temperature dependence of the reduced ordered magnetic moment (Fig. 3b) was modelled with the critical expression ${u}_{{red}}=1.06(1)\cdot {(\frac{{T}_{N}-T}{{T}_{N}})}^{0.15(1)}$ derived from mean-field theory. The obtained critical exponent *β* = 0.15(1) is close to that predicted by the 2D Ising model (*β* = 1/8 = 0.125), highlighting the 2D nature of the bct lattice and the anisotropy of 2-coordinate Co^2+^.

A corollary of this low mean magnetisation from the double-k ordered structure is that spins are only partly long-range ordered, and so substantial spin disorder remains below *T*_N_. A low-temperature paramagnetic moment of around 60% of the high-temperature effective moment is expected, assuming all the non-ordered moment components are dynamic and paramagnetic. A Curie-Weiss fit to the linear region of the reciprocal susceptibility between 2 and 15 K gave an effective moment *µ*_eff_LT_ = 3.28(2) µB and a Weiss constant of *Θ* = − 114(1) K. The effective moment is around 64% of the high-temperature value, thus supporting our magnetic model, while the negative Weiss constant reflects the dominant antiferromagnetic exchange.

The long scans shown in Fig. 3a and Supplementary Fig. S7 reveal a temperature variation of the *k*-vectors with *k* increasing from 0.566(1) at 5 K to 0.576(1) at 46 K. The temperature variation of the satellite peaks shown in Fig. 3b and the derived temperature variation of the *k*-vector (Fig. 3c) evidence a lock-in transition at around 30 K with a *k*-vector of ca. 0.565. The gradual variation of the *k*-vector at higher temperatures towards a maximum value of ca. 0.58 is attributed to a slight temperature-dependence of the balance between short-range correlations and anisotropy terms.

Previous theoretical analysis of frustrated bct lattices has predicted that the incommensurate (*k*00)/(0*k*0) magnetic ground state is stabilised when all three of the exchange interactions shown in Fig. 2a are significant, and with relative magnitudes *J*_3_ > 0.5·*J*_2_ > 0.25 *J*_1_^34^. Within this phase, the specific value of *k* is set by minimising the classical spin dispersion along (100), which yields the condition cos(π*k*) = − *J*_1_/(*J*_2_ + 2*J*_3_). The observed value of *k* = 0.57 for Ca_2_CoN_2_ therefore implies (*J*_2_ + 2*J*_3_) = 4.6·*J*_1_ confirming that the intraplanar *J*_2_ and *J*_3_ interactions dominate over the interplanar J_1_. Bct materials with (*k*00)/(0*k*0) spin density wave orders are rare, but this magnetic phase has been reported in the heavy fermion material URu_2_Si_2_ with *k* = 0.6 at low temperatures and in high magnetic fields ( ~ 35 T) adjacent to a much-studied ‘hidden order’ phase^43^. Quantum spin wave fluctuation calculations have suggested that the (*k*00)/(0*k*0) phase converts to a modulated spin liquid (MSL) around *k* ≈ 0.65-0.69 for URu_2_Si_2_^34^. Hence, the same instability may be accessible in the localised-spin magnet Ca_2_CoN_2_ by tuning physical parameters like magnetic field or through chemical doping.

DFT calculations of the exchange interactions in Ca_2_CoN_2_ (see next section and Supplementary Information for details) are in agreement with the ordering *J*_3_ > *J*_2_ > *J*_1_, and in particular highlight that all three exchange interactions are sizeable. This is in agreement with the prediction model of the bct lattice. Moreover, the calculations show that the Goodenough-Kanamori-Anderson rules apply here, with 90°-type *J*_2_ preferably ferromagnetic and 180°-type *J*_3_ preferably antiferromagnetic.

### DFT Calculations

**Fig. 5:**
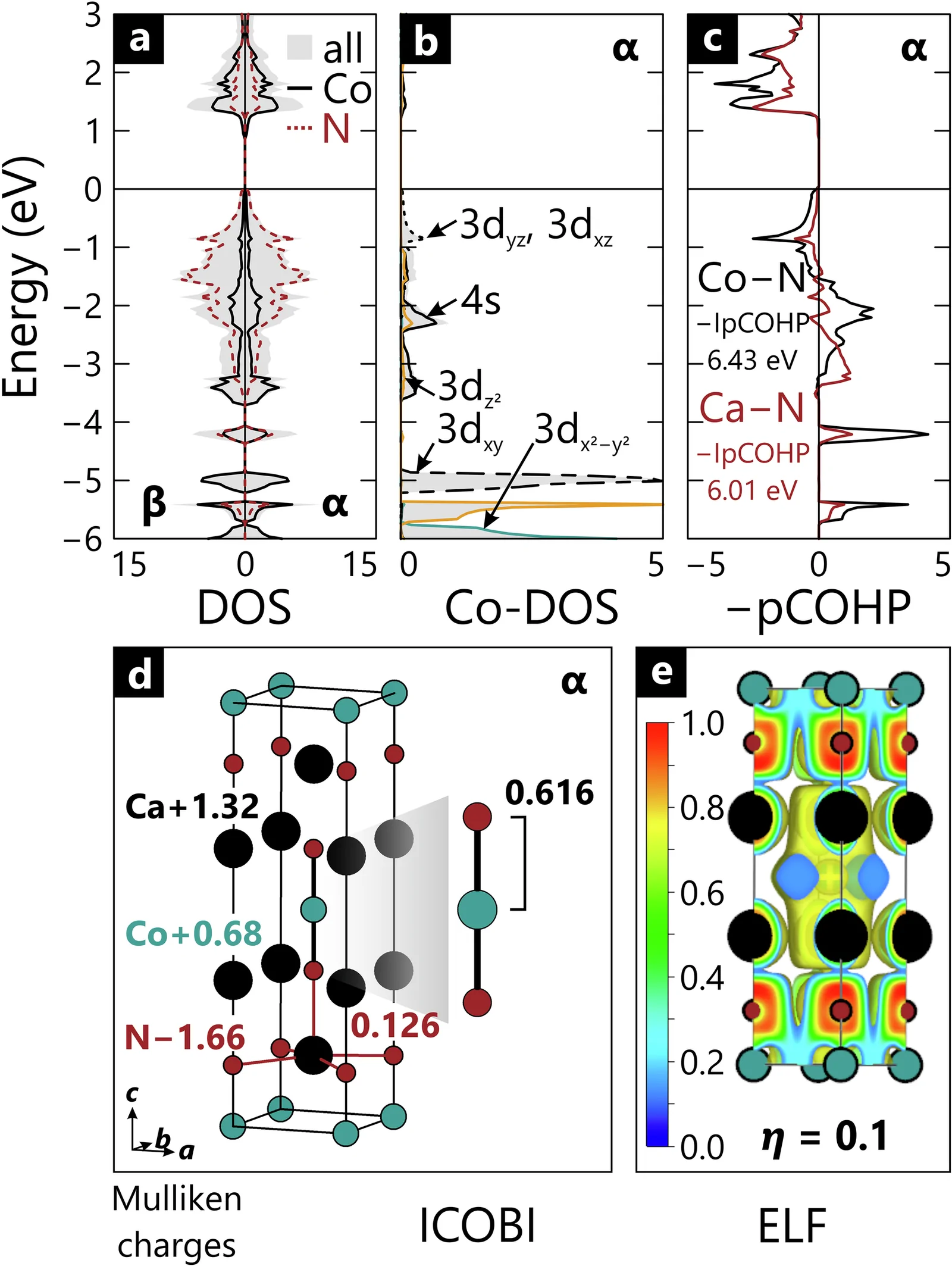
DFT calculations of Ca_2_CoN_2_. **a** spin-polarised density of states curves (DOS), (**b**) orbital-projected DOS, (**c**) projected crystal orbital Hamilton population diagram ( − pCOHP) (**d**) integrated crystal orbital bond indices (ICOBI) and (**e**) electron localisation function (ELF) of Ca_2_CoN_2_: the valence band maximum is located at 0 eV, while the orbital-projected DOS, −pCOHP and ICOBI are provided for one spin channel. The ICOBI values of both spin channels can be obtained by doubling the ICOBI included in (**d**), whereas the averaged Mulliken charges are given in units of the elementary charge *e*.

DFT-based calculations were performed to gain insight into the electronic structure and exchange interactions in Ca_2_CoN_2_ (see Supplementary Information for details). Modelsthat approximate the incommensurately modulated magnetic structure or include spin-orbit coupling were explored initially, but these models did not converge in the initial cycles of structural optimisation. The bonding analysis was therefore based on a collinear commensurate antiferromagnetic model (Fig. 5, details in Supplementary Information), as the outcome of a spin-polarised bonding analysis can slightly differ from that of a non-spin-polarised computation^44^.

To inspect possible exchange interactions arising from bonding contribution of Co, the orbital-projected densities of states (DOS) curves (Fig. 5b) were calculated. In agreement with the molecular orbital diagram of a linearly coordinated cobalt ion (Fig. 2a, right), the bands corresponding to the Co-3d_xy_ and − 3d_x2−y2_ states are lower in energy than those arising from the Co-3d_yz_ and −3d_xz_ orbitals. The modest dispersion of these bands indicates that the electrons are localised in keeping with the calculated band gap of 0.9 eV and the observed localised electron magnetic properties. Some bands originating from the Co-3d_z2_ orbitals, however, are located at a lower DOS energy region than the Co-3d_yz_ and −3d_xz_ states. This scenario originates from the admixing of the Co-3d_z2_ orbitals to Co−N σ-bonds with modest dispersion.

Under consideration of both spin channels, the ICOBI (Integrated Crystal Orbital Bond Index) values of the Ca–Co and Co–Co metal−metal contacts are lower than 0.049, which indicates that direct cation−cation exchange is negligible in Ca_2_CoN_2_. This suggests superexchange pathways *J*_1_, *J*_2_, and *J*_3_ as depicted in Fig. 2a, the relative sizes of which were estimated by comparison of total energies of a series of commensurate 2 x 2 x 1 magnetic supercell models (details in Supplementary Information, Supplementary Fig. 5). The DFT calculations indicate that intra- and interplanar exchange interactions are generally antiferromagnetic; specifically, the intralayer configuration with antiferromagnetic next-nearest neighbours is lower in energy than a nearest-neighbour antiferromagnetic model. All three exchange interaction pathways are sizeable, while the relative strength of the interactions is *J*_3_ > *J*_2_ > *J*_1_, which is in agreement with the predictions for an incommensurate ground state in the bct-type lattice (*J*_3_ > 0.5·*J*_2_ > 0.25 *J*_1_).

In summary, the outcome of the DFT calculations suggests sizable interactions as indicated by the large total energy differences between different magnetic models, while it also rules out in-plane cation−cation exchange. Therefore, longer superexchange pathways, which are suggested in Fig. 2a and can be mediated by Co−N bonding, are the likely origin of the spin density wave in Ca_2_CoN_2_.

## Discussion

The high-pressure synthesis of Ca_2_CoN_2_ demonstrates that the stabilisation of high oxidation states in late transition metal nitrides leading to localised electron magnetism can provide important new model magnetic materials. Magnetic neutron diffraction reveals a spin density wave ground state with incommensurate double-*k* magnetic ordering vectors (*k*00) and (0*k*0), where *k* = 0.5665(1) at 5 K. The incommensurability of the magnetic structure likely is a consequence of competing exchange interactions *J*_1_, *J*_2_, and *J*_3_, as predicted for the bct lattice when all three exchange pathways are significant^34^. Simultaneously, unquenched orbital momentum creates easy-axis anisotropy of Co^2+^ ions that enforces the collinearity of the transverse spin density wave. A similar Ising-like amplitude-modulated spin density wave was observed in the Co-based material Ca_3_Co_2_O_6_, which has a long-periodic modulation vector of *k* = (0 0 1.01)^45^.

The incommensurate modulation creates checkerboard-type regions of antiparallel spin orientation, leading to local antiferromagnetic alignment between next nearest neighbour moments, while nearest neighbour moments are near zero. The local pattern develops into a nanoscale ( ~ 2.5 nm) square net magnetic texture that is reminiscent of ultra-small magnetic skyrmions, despite the strict orientation along the c-axis that rules out a skyrmion ground state^39,40^. Spin-orbit coupling stabilises the emerging magnetic structure as the linear crystal field creates an anisotropy that couples the moment direction to the crystal lattice. Evidence for spin-orbit coupling is further given by an enlarged magnetic moment (*μ*_max_ = 5.00(3) μ_B_ and *μ*_max_ = 3.21(7) μ_B_ at 5 K  and 46 K, respectively, from neutron diffraction) and an effective moment (*μ*_eff_ = 5.04(1) μ_B_). The (*k*00)/(0*k*0) phase is close to a predicted instability to a modulated spin liquid phase, so further exploration of spin ordering in Ca_2_CoN_2_ under varying physical conditions or with chemical doping will be worthwhile.

In summary, this work expands the compositional and structural space of highly oxidised nitrides and demonstrates the emergence of complex magnetic order in nitrides as found in oxide-based quantum materials. The investigation of Ca_2_CoN_2_ as a model system highlights the importance of studying bct materials with weaker nearest-neighbour exchange interactions. Moreover, the study of Ca_2_CoN_2_ could be expanded to the extended family of Na_2_HgO_2_-type materials with oxide or carbide anions, which have not been widely explored as quantum magnets.

## Methods

### Preparation

#### Ca_2_CoN_2_

Samples of the title compound were prepared through a high-pressure high-temperature reaction at 8 GPa and ca. 800 °C. As starting materials, Co-metal (99.95 %, Alfa Aesar), Ca_3_N_2_ (99.9 %, Alfa Aesar) and sodium azide NaN_3_ (99.99%, Sigma-Aldrich) were used following Eq. 1. Samples were obtained as black microcrystalline powders with ca. 40 mg yield per experiment. Small amounts of an unidentified byproduct (probably Ca-rich) were detected in powder X-ray diffraction.

The high-pressure conditions were achieved with a multianvil large-volume 1000t hydraulic press built by Voggenreiter (Mainleus, Germany). The press was operated with an octahedron-within-cubes payload residing inside a Walker-type module. Co-doped tungsten carbide cubes (Hawedia GmbH, Marklkofen, Germany) with 11 mm edge-truncation served as second stage anvils and 18 mm edge Cr_2_O_3_-doped MgO-octahedra (Ceramic Substrates & Components Ltd, Lukely Works, Newport, Isle of Wight, United Kingdom) as pressure-transmitting medium. The starting materials were weighed and ground in a glovebox ( < 1 ppm O_2_/H_2_O, MBraun, Garching, Germany) and then tightly packed in a Cu-foil inlay (0.025 mm thickness, 99.9%, Thermo Scientific Chemicals), which was then placed inside an h-BN crucible that was previously treated by boiling in methanol to remove boric acid. The samples were prepared by a 120/180/60 min heating profile (ramp up, dwell, ramp down).

##### Disclaimer

Azides are highly toxic and can cause cancer while being soluble in water and stable in air. Azides can combust at elevated temperatures. Handle with care. Sodium azide decomposes to form sodium and dinitrogen.

### Diffraction

#### Powder X-ray diffraction

Powder X-ray diffraction data were obtained on a Stoe StadiP powder diffractometer (Stoe & Cie, Darmstadt, Germany) equipped with an Mo X-ray source, a Germanium(111) single crystal to filter K_α1_ radiation, and a MYTHEN 1 K Si-strip detector (Dectris, Baden, Switzerland). Samples were loaded into glass capillaries (0.5 mm outer diameter, Hilgenberg, Malsfeld, Germany). The Rietveld refinements were done with TOPAS Academic V6^46^.

Temperature-dependent powder X-ray diffraction data were collected with a Stoe StadiP powder diffractometer equipped with a graphite furnace and an image-plate position-sensitive detector. Data were collected starting at 50 °C in steps of 20 °C up to a maximum temperature of 950 °C. Samples were loaded into quartz capillaries (0.4 mm outer diameter, Hilgenberg, Malsfeld, Germany) in a glovebox and measured under exclusion of atmosphere.

#### Powder synchrotron diffraction

Synchrotron data were obtained on Ca_2_CoN_2_ on the ID22 – High Resolution Powder Diffraction Beamline of the European Synchrotron Radiation Facility at temperatures of 100 and 4 K. The diffractometer was calibrated with a Si NIST 640c reference, while the refinements were carried out with TOPAS Academic V6^1^. Pawley-type fits were used to extract the exact lattice parameters, while anisotropic peak broadening was taken into account using a Stephens model (reflections with high l-values showed broadening in line with a layered compound prepared under high-pressure conditions)^47,48^.

#### Powder Neutron diffraction

Powder diffraction data of Ca_2_CoN_2_ were collected on the high-intensity powder diffractometer D20 of the Institut Laue-Langevin (Grenoble, France). High-resolution data using a take-off angle of 90° and a wavelength of 1.54 Å were collected at 295 , 100, and 5 K, while long scans with high-flux at a take-off angle of 42° and a wavelength of 2.4 Å were collected at 5, 46, and 100 K. Temperature-dependent measurements (short scans) were collected from 7 K to 98 K. Rietveld refinement was done using the programme Fullprof ^35^. Crystal structures were visualised using VESTA^49^.

Incommensurate Magnetic Crystallographic Information Files were created with the ISODISTORT web-based tool (https://iso.byu.edu/isodistort.php)^36,37^. Commensurate approximations were created with the Bilbao Crystallographic Server function STRCONVERT (https://cryst.ehu.es/cgi-bin/cryst/programs/mcif2vesta/index.php)^50–52^.

### Physical Properties Measurements

Magnetic properties of Ca_2_CoN_2_ were measured with a vibrating sample magnetometer option for a Quantum Design Physical Property Measurement System (PPMS, San Diego, CA, USA). Susceptibility data were recorded in a field of 30 kOe in a temperature range between 2 and 300 K. Field-dependent measurements were conducted at 2 and 300 K in fields between ± 50 kOe. The sample was placed inside polyethylene capsules of known diamagnetic contribution.

### DFT Calculations

Electronic structure calculations were performed to gain insight into the nature of chemical bonding as well as the exchange interactions in Ca_2_CoN_2_. Under consideration of the experimentally determined magnetic structure, models which approximated an incommensurately modulated magnetic structure or included spin-orbit coupling were also probed; however, the calculations related to these models did not converge in the initial cycles of the structural optimisations. Therefore, the chemical bonding analysis was accomplished based on a collinear antiferromagnetic starting model where the computed magnetic moment (*μ*_Co_ = 2.4 μ_B_, T = 0 K) on average approaches the amplitudes which were determined for the magnetic moment at 5 K. This well reproduces the lattice parameters and atomic positions after the initial cycles of structural optimisation. To explore the nature of the exchange interactions, the total energies for different magnetic configurations were also evaluated, which were modelled based on a 2 × 2 × 1 superstructure of the experimentally determined Ca_2_CoN_2_ structure model (Supplementary Table S1 and Supplementary Fig. S5).

All electronic structure calculations were accomplished by using the projector augmented wave (PAW) method^53^ within the Vienna ab initio simulation package^54–58^ (VASP). These computations also included full optimisations of the lattice parameters and atomic positions, while correlation and exchange were described by the generalised gradient approximation of Perdew, Burke and Ernzerhof^59^ (GGA − PBE). In the initial cycles of refinement, the size and the magnetic moments were not well reproduced for the cobalt atoms due to an inadequate description of the correlations related to the 3 d states. Therefore, a Hubbard U parameter (*U*_eff_ = 5 eV) that was established based on a preliminary screening was included in all spin-polarised computations. The energy cut-off of the plane waves was set to 500 eV, while sets of 20 × 20 × 5 and 20 × 20 × 12 k-points were employed to sample the first Brillouin zones of the structure models related to the Ca_2_CoN_2_ structure model and the superstructures, respectively. All computations were considered converged, as the energy difference between two iterative steps fell below 10^−8^ eV (10^−6^ eV) of the electronic (ionic) relaxation.

In order to get an insight into the distributions of the valence electrons in Ca_2_CoN_2_, chemical bonding analysis were completed based on the projected crystal orbital Hamilton populations^60,61^ (pCOHP), the integrated crystal orbital bond indices^62^ (ICOBI) and the Mulliken^63^ charges. Because the aforementioned bonding descriptors are constructed based on local basis sets whose nature is in stark contrast to those of the plane waves employed in the DFT-based computations, the results of the plane wave-based calculations had to be transferred to all Slater-type orbitals. These projections were accomplished with the aid of the Local Orbital Basis Suite Towards Electronic-Structure Reconstruction^64,65^ (LOBSTER) code. The spin-polarised DOS curves and −pCOHP diagrams were visualised by using the wxdragon^66^ code, while representations showing the crystal structure were created utilising the programmes Diamond and VESTA^49,67^. Furthermore, the electron localisation function^68–70^ (ELF) was computed by means of the aforementioned VASP code and visualised with the aid of the programme VESTA^5,49^.

## Supplementary information

Supplementary Information

Transparent Peer Review file

## Source data

Source Data 1

Source Data 2

Source Data 3

Source Data 4

## Data Availability

The diffraction and magnetisation data generated in this study are available on the Open Access LMU repository (https://doi.org/10.5282/ubm/data.853). Until deposition is complete, the data are available from the corresponding author (S.D. Kloß, simon.kloss@cup.lmu.de) upon request. Crystallographic data for Ca_2_CoN_2_ have been deposited with the joint CCDC/FIZ Karlsruhe deposition service under deposition numbers CSD-2528420, 2528421, 2528422 and 2528423, and are available free of charge via https://www.ccdc.cam.ac.uk/structures/. The following Magnetic Crystallographic Information Files (mCIFs) are provided as source data with this article: incommensurate double-k model, commensurate double-k approximation (7 × 7 superstructure), incommensurate single-k model, and commensurate single-k model (7 × 7 superstructure). Source data are provided in this paper.

## References

[1] Tranquada, J. M., Buttrey, D. J., Sachan, V. V. & Lorenzo, J. E. Simultaneous ordering of holes and spins in La2NiO4.125. *Phys. Rev. Lett.* **73**, 1003–1006 10.1103/PhysRevLett.73.1003 (1994). 10057595

[2] Kim, B. J. et al. Novel Jeff=1/2 Mott state induced by relativistic spin-orbit coupling in Sr2IrO4. *Phys. Rev. Lett.* **101**, 076402 10.1103/PhysRevLett.101.076402 (2008). 18764560

[3] Maeno, Y. et al. Superconductivity in a layered perovskite without copper. *Nature* **372**, 532–534 10.1038/372532a0 (1994).

[4] Petsch, A. N. et al. High-energy spin waves in the spin-1 square-lattice antiferromagnet La2NiO4.* Phys. Rev. Res*.** 5**, 033113 10.1103/PhysRevResearch.5.033113 (2023).

[5] Coldea, R. et al. Spin waves and electronic interactions in La2CuO4. *Phys. Rev. Lett.* **86**, 5377–5380 10.1103/PhysRevLett.86.5377 (2001). 11384502

[6] Headings, N. S., Hayden, S. M., Coldea, R. & Perring, T. G. Anomalous high-energy spin excitations in the high-Tc superconductor-parent antiferromagnet La2CuO4. *Phys. Rev. Lett.* **105**, 247001 10.1103/PhysRevLett.105.247001 (2010). 21231553

[7] Shrestha, S. et al. Emergent interlayer magnetic order via strain-induced orthorhombic distortion in the 5d Mott insulator Sr2IrO4.* Phys. Rev. B*** 105**, 10.1103/PhysRevB.105.L100404 (2022).

[8] Hücker, M., Klauss, H. H. & Büchner, B. Strong dependence of the interlayer coupling on the hole mobility in antiferromagneticLa2−xSrxCuO4 (x<0.02).* Phys. Rev. B*** 70**, 10.1103/PhysRevB.70.220507 (2004).

[9] Yamada, K. et al. Magnetic structure and weak ferromagnetism of La2NiO4+δ. *Phys. C* **191**, 15–22 10.1016/0921-4534(92)90625-m (1992).

[10] Ebel, T., Wachtmann, K. H. & Jeitschko, W. Magnetic properties of the uranium transition metal carbides U2TC2 (T = Ru, Os, Rh, Ir and Pt). *Solid State Commun* **97**, 815–819 10.1016/0038-1098(95)00516-1 (1996).

[11] Haines, H. R. & Potter, P. E. Ternary compounds of Uranium–Carbon and the group VIII A elements. *Nature* **221**, 1238–1239 10.1038/2211238b0 (1969).

[12] Rieck, H. & Hoppe, R. Über Oxoniccolate(II) der Alkalimetalle:K2NiO2, Rb2NiO2 und Cs2NiO2. *Z. Anorg. Allg. Chem* **400**, 311–320 10.1002/zaac.19734000310 (1973).

[13] Đuriš, K. & Jansen, M. Cs2NiO2 Revisited. Crystal structure and magnetic properties. *Z. Naturforsch. B* **67**, 57–60 10.1515/znb-2012-0110 (2012).

[14] Chern, M. Y. & DiSalvo, F. J. Synthesis, structure, and properties of Ca2ZnN2. *J. Solid State Chem.* **88**, 528–533 10.1016/0022-4596(90)90250-2 (1990).

[15] Yamane, H. & DiSalvo, F. J. Synthesis and crystal structure of Sr2ZnN2 and Ba2ZnN2. *J. Solid State Chem.* **119**, 375–379 10.1016/0022-4596(95)80055-t (1995).

[16] Kloß, S. D. & Attfield, J. P. Low-dimensional magnetism in calcium nitridonickelate(II) Ca2NiN2. *Chem. Commun.* **57**, 10427–10430 10.1039/d1cc04001d (2021). 34549238

[17] Wakeham, N. et al. Magnetism and superconductivity inU2PtxRh1−xC2.* Phys. Rev. B*** 91**, 024408 (2015).

[18] Machado, A. J. S., Grant, T. & Fisk, Z. Th2NiC2: a low density of states superconductor.* Supercond. Sci. Technol*.** 25**, 045010 (2012).

[19] Wang, J.-P. Environment-friendly bulk Fe16N2 permanent magnet: Review and prospective. *J. Magn. Magn. Mater.* **497**, 165962–165962 10.1016/j.jmmm.2019.165962 (2020).

[20] Rimmler, B. H., Pal, B. & Parkin, S. S. P. Non-collinear antiferromagnetic spintronics. *Nat. Rev. Mater.* **10**, 109–127 10.1038/s41578-024-00706-w (2024).

[21] Trócoli, R. et al. MnTa2N4: A ternary nitride spinel with a strong magnetic frustration. *Chem. Mater.* **34**, 6098–6107 10.1021/acs.chemmater.2c01240 (2022).

[22] Kloß, S. D., Weidemann, M. L. & Attfield, J. P. Preparation of bulk-phase nitride perovskite LaReN3 and topotactic reduction to LaNiO2 -Type LaReN2. *Angew. Chem., Int. Ed.* **60**, 22260–22264 10.1002/anie.202108759 (2021). 34355842

[23] Weidemann, M. et al. High-pressure synthesis of Ruddlesden–Popper nitrides. *Nat. Chem.* **16**, 1723–1731 10.1038/s41557-024-01558-1 (2024). 38918580

[24] Kloß, S. D. et al. Preparation of iron(IV) nitridoferrate Ca4FeN4 through azide-mediated oxidation under high-pressure conditions. *Nat. Commun.* **12**, 571 10.1038/s41467-020-20881-y (2021). PMC7835361 33495442

[25] Yuan, Y., Yang, M., Kloß, S. D. & Attfield, J. P. A new family of high oxidation state antiperovskite nitrides: La3MN5 (M=Cr, Mn and Mo). *Angew. Chem., Int. Ed.* **63**, e202405498 10.1002/anie.202405498 (2024). 38651652

[26] Meskine, H. & Satpathy, S. Electronic structure and magnetism in sodium nickelate: Density-functional and model studies. *Phys. Rev. B*** 72**, 224423 10.1103/PhysRevB.72.224423 (2005).

[27] Bendyna, J. K., Höhn, P. & Kniep, R. Crystal structure of pentacalcium bis(dinitridocobaltate(I)), Ca_5_[CoN_2_]_2_, and a note on “Ca_3_CoN_3_”. *Z. Kristallogr. New Cryst. Struct.* **222**, 165–166 10.1524/ncrs.2007.0068 (2007).

[28] Ballé, T. J., Zangeneh, Z., Hozoi, L., Jesche, A. & Höhn, P. Ferromagnetic ordering of linearly coordinated Co ions in LiSr2[CoN2]. *Phys. Rev. B* **99**, 1–9 10.1103/PhysRevB.99.094422 (2019).

[29] Bunting, P. C. et al. A linear cobalt(II) complex with maximal orbital angular momentum from a non-Aufbau ground state.* Science*** 362**, 10.1126/science.aat7319 (2018). 30442763

[30] Power, P. P. Stable two-coordinate, open-shell (d1-d9) transition metal complexes. *Chem. Rev.* **112**, 3482–3507 10.1021/cr2004647 (2012). 22480221

[31] Figgis, B. N. & Hitchman, M. A.* Ligand Field Theory and Its Applications*. (Wiley & Sons Ltd, 2000).

[32] Hitchman, M. A., Stratemeier, H. & Hoppe, R. Electronic and Raman spectra of the linear dioxonickelate(2-) ion in dipotassium dioxonickelate(2-). *Inorg. Chem.* **27**, 2506–2510 10.1021/ic00287a026 (1988).

[33] Bryan, A. M., Merrill, W. A., Reiff, W. M., Fettinger, J. C. & Power, P. P. Synthesis, structural, and magnetic characterization of linear and bent geometry cobalt(II) and nickel(II) amido complexes: evidence of very large spin-orbit coupling effects in rigorously linear coordinated Co2+. *Inorg. Chem.* **51**, 3366–3373 10.1021/ic2012414 (2012). 22360619

[34] Farias, C. et al. Spin liquid versus long-range magnetic order in the frustrated body-centered-tetragonal lattice.* Phys. Rev. B*** 94**, 134420 10.1103/PhysRevB.94.134420 (2016).

[35] Rodríguez-Carvajal, J. Recent advances in magnetic structure determination by neutron powder diffraction. *Phys. B* **192**, 55–69 10.1016/0921-4526(93)90108-i (1993).

[36] Campbell, B. J., Stokes, H. T., Tanner, D. E. & Hatch, D. M. ISODISPLACE: a web-based tool for exploring structural distortions. *J. Appl. Crystallogr.* **39**, 607–614 10.1107/s0021889806014075 (2006).

[37] Stokes, H. T., Campbell, B. J. & Hatch, D. M. Order parameters for phase transitions to structures with one-dimensional incommensurate modulations. *Acta Crystallogr. A* **63**, 365–373 10.1107/S0108767307024890 (2007). 17570899

[38] Fitch, A. et al. ID22 - the high-resolution powder-diffraction beamline at ESRF. *J. Synchrotron Radiat.* **30**, 1003–1012 10.1107/S1600577523004915 (2023). PMC10481261 37462688

[39] Tokura, Y. & Kanazawa, N. Magnetic skyrmion materials. *Chem. Rev.* **121**, 2857–2897 10.1021/acs.chemrev.0c00297 (2021). 33164494

[40] Hirschberger, M. et al. Skyrmion phase and competing magnetic orders on a breathing kagome lattice. *Nat. Commun.* **10**, 5831 10.1038/s41467-019-13675-4 (2019). PMC6930224 31874953

[41] Smura, C. F. et al. High-spin cobalt(II) ions in square planar coordination: structures and magnetism of the oxysulfides Sr2CoO2Cu2S2 and Ba2CoO2Cu2S2 and their solid solution. *J. Am. Chem. Soc.* **133**, 2691–2705 10.1021/ja109553u (2011). 21302927

[42] Jauch, W., Reehuis, M., Bleif, H. J., Kubanek, F. & Pattison, P. Crystallographic symmetry and magnetic structure of CoO.* Phys. Rev. B*** 64**, 052102 10.1103/PhysRevB.64.052102 (2001).

[43] Knafo, W. et al. Field-induced spin-density wave beyond hidden order in URu2Si2. *Nat Commun* **7**, 13075 10.1038/ncomms13075 (2016). PMC5080431 27762260

[44] Landrum, G. A. & Dronskowski, R. The orbital origins of magnetism: from atoms to molecules to ferromagnetic alloys. *Angew. Chem. Int. Ed.* **39**, 1560–1585 10.1002/(sici)1521-3773(20000502)39:9<1560::Aid-anie1560>3.0.Co;2-t (2000). 10820443

[45] Agrestini, S. et al. Nature of the magnetic order in Ca3Co2O6. *Phys. Rev. Lett.* **101**, 097207 10.1103/PhysRevLett.101.097207 (2008). 18851656

[46] Coelho, A. A. TOPAS and TOPAS-Academic: an optimization program integrating computer algebra and crystallographic objects written in C++. *J. Appl. Crystallogr.* **51**, 210–218 10.1107/s1600576718000183 (2018).

[47] Stephens, P. W. Phenomenological model of anisotropic peak broadening in powder diffraction. *J. Appl. Crystallogr.* **32**, 281–289 10.1107/s0021889898006001 (1999).

[48] Pawley, G. S. Unit-cell refinement from powder diffraction scans. *J. Appl. Crystallogr.* **14**, 357–361 10.1107/s0021889881009618 (1981).

[49] Momma, K. & Izumi, F. VESTA 3 for three-dimensional visualization of crystal, volumetric and morphology data. *J. Appl. Cryst.* **44**, 1272–1276 10.1107/S0021889811038970 (2011).

[50] Aroyo, M. I. et al. Crystallography online: Bilbao Crystallographic Server. *Bulg. Chem. Commun.* **43**, 183–197 (2011).

[51] Aroyo, M. I. et al. Bilbao Crystallographic Server: I. Databases and crystallographic computing programs. *Zeitschrift für Kristallographie Crystalline Materials* **221**, 15–27 10.1524/zkri.2006.221.1.15 (2006).

[52] Aroyo, M. I., Kirov, A., Capillas, C., Perez-Mato, J. M. & Wondratschek, H. Bilbao Crystallographic Server. II. Representations of crystallographic point groups and space groups. *Acta Crystallogr. A* **62**, 115–128 10.1107/S0108767305040286 (2006). 16489249

[53] Blöchl, P. E. Projector augmented wave method. *Phys. Rev. B Condens. Matter Mater. Phys.* **50**, 17953–17979 10.1103/PhysRevB.50.17953 (1994). 9976227

[54] Kresse, G. & Hafner, J. Ab initio molecular dynamics for liquid metals. *Phys. Rev. B Condens. Matter Mater. Phys.* **47**, 558–561 10.1103/PhysRevB.47.558 (1993). 10004490

[55] Kresse, G. & Furthmüller, J. Efficiency of ab-initio total energy calculations for metals and semiconductors using a plane-wave basis set. *Comput. Mater. Sci.* **6**, 15–50 10.1016/0927-0256(96)00008-0 (1996). 9984901

[56] Kresse, G. & Furthmüller, J. Efficient iterative schemes for ab initio total-energy calculations using a plane-wave basis set. *Phys. Rev. B Condens. Matter Mater. Phys.* **54**, 11169–11186 10.1103/PhysRevB.54.11169 (1996). 9984901

[57] Kresse, G. & Joubert, D. From ultrasoft pseudopotentials to the projector augmented-wave method. *Phys. Rev. B Condens. Matter Mater. Phys.* **59**, 1758–1775 10.1103/PhysRevB.59.1758 (1999).

[58] Kresse, G., Marsman, M. & Furthmüller, J.* Vienna Ab Initio Simulation Package (VASP)*,* The Guide*. (2014).

[59] Perdew, J. P., Burke, K. & Ernzerhof, M. Generalized gradient approximation made simple. *Phys. Rev. Lett.* **77**, 3865–3868 10.1103/PhysRevLett.77.3865 (1996). 10062328

[60] Dronskowski, R. & Bloechl, P. E. Crystal orbital Hamilton populations (COHP). Energy-resolved visualisation of chemical bonding in solids based on density-functional calculations. *J. Phys. Chem.* **97**, 8617–8624 10.1021/j100135a014 (1993).

[61] Deringer, V. L., Tchougréeff, A. L. & Dronskowski, R. Crystal orbital Hamilton population (COHP) analysis as projected from plane-wave basis sets. *J. Phys. Chem. A* **115**, 5461–5466 10.1021/jp202489s (2011). 21548594

[62] Müller, P. C., Ertural, C., Hempelmann, J. & Dronskowski, R. Crystal orbital bond index: covalent bond orders in solids. *J. Phys. Chem. C* **125**, 7959–7970 10.1021/acs.jpcc.1c00718 (2021).

[63] Ertural, C., Steinberg, S. & Dronskowski, R. Development of a robust tool to extract Mulliken and Löwdin charges from plane waves and its application to solid-state materials. *RSC Adv.* **9**, 29821–29830 10.1039/C9RA05190B (2019). PMC9071923 35531548

[64] Maintz, S., Deringer, V. L., Tchougréeff, A. L. & Dronskowski, R. Analytic projection from plane-wave and PAW wavefunctions and application to chemical-bonding analysis in solids. *J. Comput. Chem.* **34**, 2557–2567 10.1002/jcc.23424 (2013). 24022911

[65] Maintz, S., Deringer, V. L., Tchougréeff, A. L. & Dronskowski, R. LOBSTER: A tool to extract chemical bonding from plane-wave based DFT. *J. Comput. Chem.* **37**, 1030–1035 10.1002/jcc.24300 (2016). PMC5067632 26914535

[66] Eck, B.* wxDragon 2.2.3*. (RWTH Aachen University, Aachen, Germany, 2020).

[67] Diamond version 4.6.8 (Crystal Impact GbR, Bonn, Germany, 2022).

[68] Savin, A., Nesper, R., Wengert, S. & Fässler, T. F. ELF: The electron localization function. *Angew. Chem. Int. Ed.* **36**, 1808–1832 10.1002/anie.199718081 (1997).

[69] Becke, A. D. & Edgecombe, K. E. A simple measure of electron localization in atomic and molecular systems. *J. Chem. Phys.* **92**, 5397–5403 10.1063/1.458517 (1990).

[70] Silvi, B. & Savin, A. Classification of chemical bonds based on topological analysis of electron localization functions. *Nature* **371**, 683–686 10.1038/371683a0 (1994).

