# Supplementary material for: Complex magnetism in a transition metal nitride
Source: Nat Commun. 2026 Nov 2;17:11331. doi: 10.1038/s41467-026-78995-8 (PMC13728190; doi:10.1038/s41467-026-78995-8)
Supplement: Supplementary file 1 [file 41467_2026_78995_MOESM1_ESM.pdf]

Supplementary Information

Complex magnetism in a transition metal nitride

Table of Contents

Supporting data for powder X-ray and neutron diffraction 2

Temperature dependent powder X-ray diffraction 5

Supporting data for magnetic measurements 6

Supporting data for magnetic powder neutron diffraction measurements 7

Supporting data for DFT-based Calculations 8

Synchrotron X-ray Diffraction 12

Additional Information on Magnetic Powder Neutron Diffraction 15

References 15

Supporting data for powder X-ray and neutron diffraction

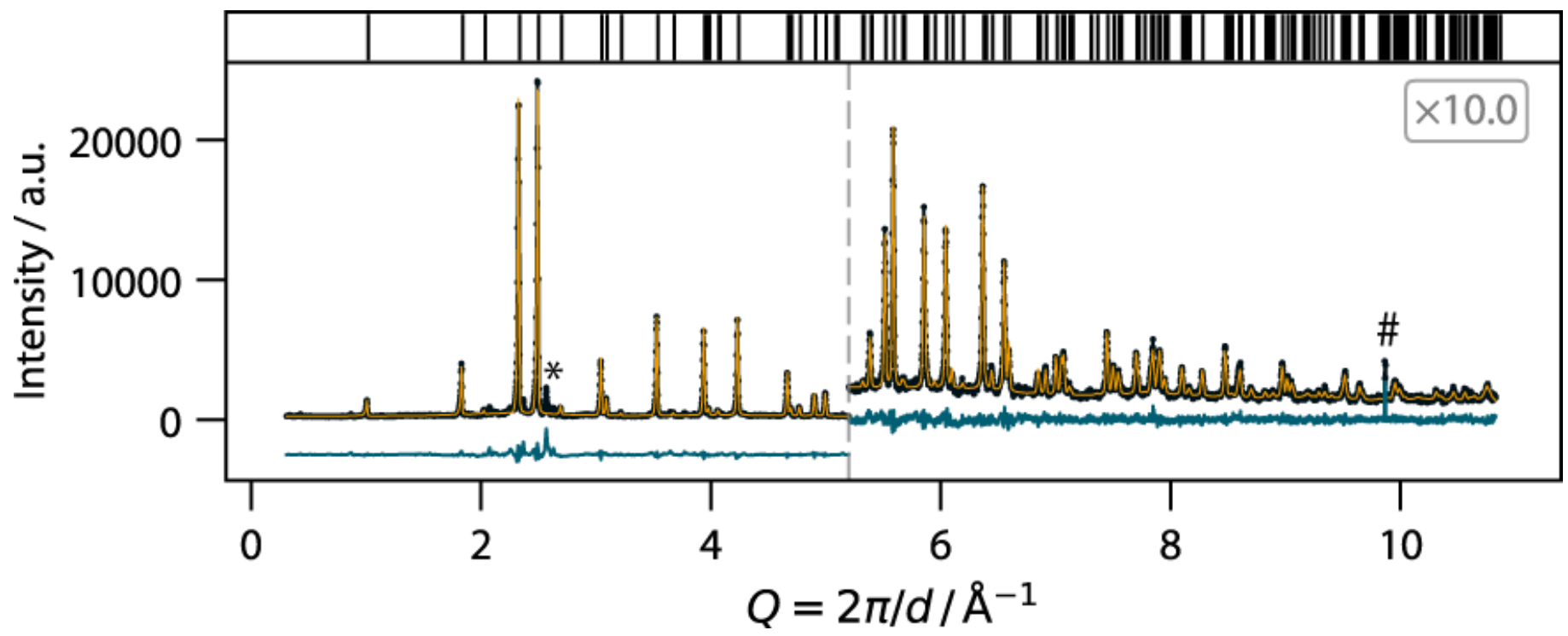

Supplementary Figure 1: Rietveld refinement of powder X-ray diffraction data obtained on Ca_2CoN_2. Data as black points, fit as orange line, difference curve as blue line, Bragg reflection positions as black vertical lines. Unidentified byproduct marked by *, faulty detector cell marked by #. Right side of dashed grey line shows 10x magnified area of low intensity region.

Table S1: Crystallographic data for Ca_2CoN_2 recorded on an in-house powder X-ray diffractometer and the D20 beamline of the Institut Laue-Langevin.

**Crystal data**				
Formula	Ca_2CoN_2			
Formula mass, g·mol^{-1}	167.1			
Crystal system	tetragonal			
Space group	*I4/mmm* (no. 139)			
Dataset	PXRD 300 K	PND 295 K	PND 100 K	PND 5 K
Cell parameters, Å	*a* = 3.5509(2)	*a* = 3.57175(9)	*a* = 3.56344(8)	*a* = 3.56232(9)
	c = 12.3465(6)	*c* = 12.4127(6)	*c* = 12.4107(5)	*c* = 12.4117(6)
Cell volume, Å^3	155.68(2)	158.35(1)	157.59(1)	157.51(1)
Z	2			
F(000)	162			
Calc. density, g·cm^{-3}	3.565	3.504	3.521	3.523
**Data collection**				
Diffractometer	Stoe & Cie StadiP	D20, Institut Laue-Langevin		
Radiation	Mo-K$_{\alpha1}$	Constant Wavelength Neutron, λ = 1.54 Å		
Temperature, K	300	295	100	5
Q-range, Å^{-1}	0.31, 10.82	0.71, 7.77		
Measured reflections	161	68		
Absorption correction	Cylindrical			
**Refinement**				
GOF	2.6	5.9	6.2	6.6
R_p, R_{wp}, R_{exp}	8.0, 13.2, 5.0	2.6, 3.7, 0.6	2.8, 3.9, 0.6	3.0, 4.2, 0.6
R_{Bragg}	1.75	3.1	3.1	3.1

Table S2: Atom positions, occupancies, displacement parameter of an equivalent sphere, Wyckoff positions and site symmetries for all recorded powder diffraction data.

Atom	*x*	*y*	*z*	Occupancy	U_{eq} / Å^2	Wyckoff	Site Symm.
**PXRD 300 K**							
Co1	0	0	0	1	0.0115(7)	2a	4/*mmm*
Ca1	0	0	0.34396(14)	1	0.0065(6)	4e	4*mm*
N1	0	0	0.1455(5)	1	0.0103(19)	4e	4*mm*
**PND 295 K**							
Co1	0	0	0	1	0.0063(15)	2a	4/*mmm*
Ca1	0	0	0.3442(3)	1	0.0135(8)	4e	4*mm*
N1	0	0	0.14379(17)	1	0.0159(4)	4e	4*mm*
**PND 100 K**							
Co1	0	0	0	1	0.0018(13)	2a	4/*mmm*
Ca1	0	0	0.3440(3)	1	0.0086(7)	4e	4*mm*
N1	0	0	0.14404(16)	1	0.0116(4)	4e	4*mm*
**PND 5 K**							
Co1	0	0	0	1	0.0008(14)	2a	4/*mmm*
Ca1	0	0	0.3439(3)	1	0.0074(7)	4e	4*mm*
N1	0	0	0.14390(16)	1	0.0113(4)	4e	4*mm*

Table S3: Anisotropic displacement parameters for the refinements of all recorded powder diffraction data.

Atom	U_{11} / Å^2	U_{22} / Å^2	U_{33} / Å^2	U_{12} / Å^2	U_{13} / Å^2	U_{23} / Å^2
**PXRD 300 K**						
Co1	0.0144(9)	0.0144(9)	0.0056(15)	0	0	0
Ca1	0.0056(6)	0.0056(6)	0.0083(17)	0	0	0
N1	0.007(2)	0.007(2)	0.017(5)	0	0	0
**PND 295 K**						
Ca1	0.0123(10)	0.0123(10)	0.016(2)	0	0	0
N1	0.0134(7)	0.0134(7)	0.0208(9)	0	0	0
**PND 100 K**						
Ca1	0.0074(9)	0.0074(9)	0.0110(18)	0	0	0
N1	0.0082(7)	0.0082(7)	0.0185(8)	0	0	0
**PND 5 K**						
Ca1	0.0068(9)	0.0068(9)	0.0086(18)	0	0	0
N1	0.0077(7)	0.0077(7)	0.0185(9)	0	0	0

Table S4: Observed interatomic distances Ca–N and Co–N for all recorded powder diffraction data.

**PXRD 300 K**	***d*** **/ Å**	**PND 295 K**	***d*** **/ Å**	**PND 100 K**	***d*** **/ Å**	**PND 5 K**	***d*** **/ Å**
Co1–N1	1.796(6)	Co1–N1	1.785(2)	Co1–N1	1.788(2)	Co1–N1	1.786(2)
	1.796(6)		1.785(2)		1.788(2)		1.786(2)
Ca1–N1	2.5142(4)	Ca1–N1	2.5300(3)	Ca1–N1	2.5241(3)	Ca1–N1	2.5235(3)
	2.5142(4)		2.5300(3)		2.5241(3)		2.5235(3)
	2.450(7)		2.488(5)		2.482(5)		2.482(5)
	2.5142(4)		2.5300(3)		2.5241(3)		2.5235(3)
	2.5142(4)		2.5300(3)		2.5241(3)		2.5235(3)

Temperature dependent powder X-ray diffraction

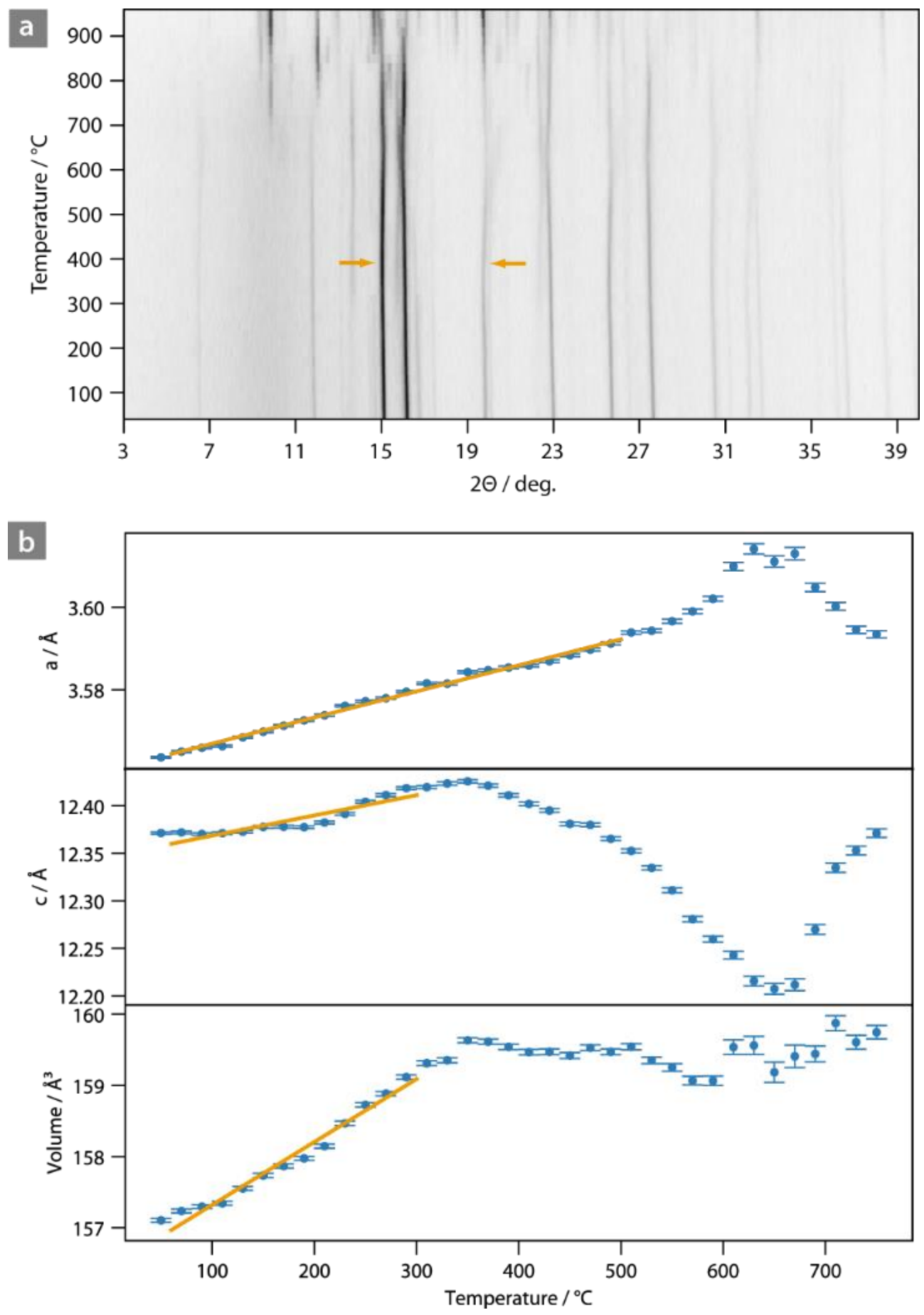

Supplementary Figure 2: a Temperature dependent powder X-ray diffraction data collected on Ca_2CoN_2 in the range from 50 to 950 °C. Reflections shifting to smaller *d*-spacing are marked with orange arrows. **b** Temperature-dependent evolution of lattice parameters, highlighting the onset of structural changes starting at ca. 300 °C and decomposition setting in at around 600 °C. Fits of linear expansion are marked as orange lines.

Supporting data for magnetic measurements

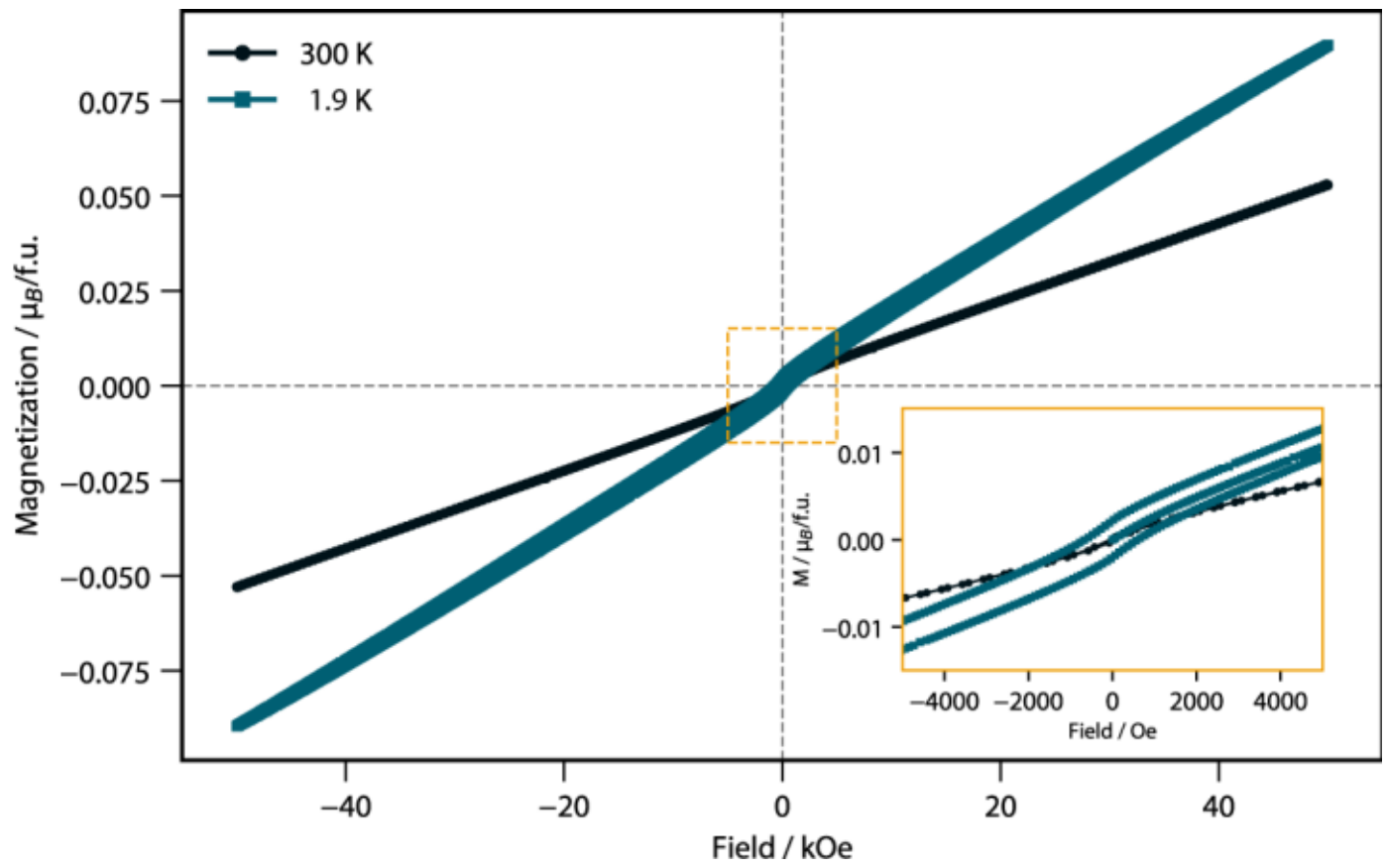

Supplementary Figure 3: Field-dependent magnetisation of Ca_2CoN_2 obtained at 300 and 1.9 K in fields of ±50 kOe. The inset shows a region ±5 kOe highlighting a small ferromagnetic impurity that is visible at 1.9 and 300 K. As the T_C seems to be above room temperature, small amounts of unreacted Co-metal are a likely source.

Supporting data for magnetic powder neutron diffraction measurements

Single k = (0 0.57 0) magnetic structure.

Magnetic symmetry analysis using the program BASIREPS for Co on (0 0 0) with k = [0, 0.57, 0] returns three irreducible representations having each a single basis vector along either of the principle axis, *a*, *b*, or *c*.[1] Only an incommensurate magnetic structure (Fig. 3a) where the magnetic moments point along the *c*-direction is able to refine the data (Fig. 3b); magnetic space group *Immm*.1'(0,0,g)s00s.[2] The magnetic structure can be described as a single sine-wave running in direction of *b* with the magnetic moment $\mu(y) = \mu_{max} \cdot \sin(2\pi \cdot \frac{q \cdot y}{b}) = \mu_{max} \cdot \sin(2\pi \cdot q \cdot n)$, with *y* being the position of the Co-atoms and n = the number of the unit cells along b. The maximum amplitudes were determined with μ_{max} (5 K) = 3.53(2) μ_B and μ_{max}(46 K) = 2.27(5) μ_B, while the wavelength of the modulation is $\lambda_{5K} = b/q_{5K} \approx 6.25$ Å. The latter indicates that a seven-fold superstructure (Fig. 3a bottom) can serve as a commensurate approximation with $7b/\lambda_{5K} = 3.99 \approx$ integer. A double-*k* magnetic structure could arise by magnetic domains with k = [0, 0.57, 0] and k = [0.57, 0, 0].

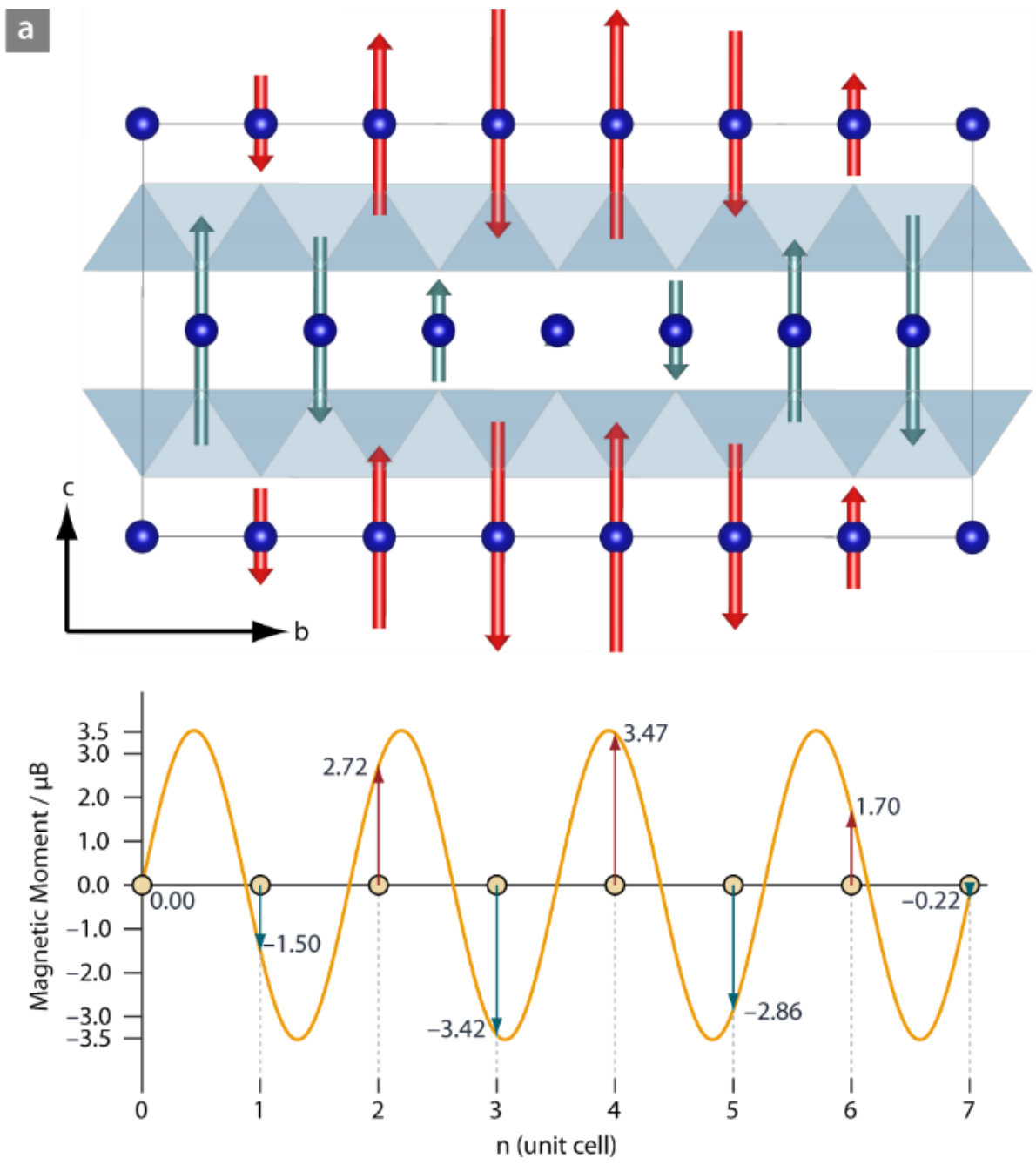

Supplementary Figure 4. a Magnetic structure of Ca_2CoN_2 (top) represented by a 7-fold superstructure along b. The bottom picture shows the modulation of the magnetic moment of one string of Co-atoms by the vector k = (0, 0.57, 0) for n = 7 unit cells. Along the a-direction (perpendicular to paper-plane), the magnetic moments align ferromagnetically. The shift of 1/2*b* between Co atoms in different planes (red vs. blue magnetic moment vectors) lead to a phase shift of $\pi \cdot k$.

Supporting data for DFT-based Calculations

Additional discussion of the bonding situation

The population analysis based on the Mulliken charges (Fig. 5d of the main text) reveals that calcium serves as a valence electron donor, resulting in polar Ca–N interactions with a minor degree of covalency mirrored by the modest integrated crystal orbital bond index (ICOBI) values. The Co–N interactions are covalent as indicated by Mulliken charges as well as ICOBI and integrated −pCOHP/bond values. The calculated Ca–N and Co–N interactions are in line with the classic picture of a nitridometallate and highlight that a simple ionic picture represented by the formula $(Ca^{2+})_2(Co^{2+})(N^{3-})_2$ is insufficient.

The Co-3d_{yz} and -3d_{xz} bands exhibit a broader dispersion than the remaining Co-3d bands, as they combine with the N-2p orbitals to partially occupied π-orbitals. Accordingly, the large Co−N ICOBI of both spin components (1.232) exceeds values (~ 1) encountered for two-electron−two-centre bonds, while the N−Co−N three-centre ICOBI of both spin channels (−0.261) is evident for multicentre bonding.[3] This conclusion is supported by an analysis of the electron localization function localization because domains begin to appear around the $[CoN_2]$ units for $\eta < 0.22$ (Fig. 5e). The notion of multiple bonding in Ca_2CoN_2 is further backed by the rather short Co–N distances (d_{Co-N} = 1.796(7) Å) when compared to previously reported Co−N single bonds (> 1.90 Å).[4]

Additional discussion of the exchange interactions

DFT-based calculations indicate that large interatomic distances between Co-sites prevent direct metal-metal exchange, so that exchange pathways are likely via Ca–N layers. Moreover, DFT suggests strong Co–N dπ–pπ bonding, which can enhance the exchange strength as suggested based on previous reports on quantification of Anderson superexchange via angular overlap modelling.[5] The strength of these interactions is reflected in the T_N of 54 K, which is probably lower than the true exchange strength since the larger Weiss-constant indicates a frustration parameter of f = 3.6. We propose that spin information is transmitted in die Ca–N layers via longer Co–N–Ca–N–Co superexchange pathways similar to nickelate $NaNiO_2$.[6,7] In RP-type materials rocksalt layers equivalent to the Ca–N layers also transmit spin information and are, for example, responsible for three-dimensional magnetic ordering. Interestingly, the intralayer coupling in Ca_2CoN_2 is also via these Ca–N layers, while in analogous RP-type oxides, the intralayer interaction is within the M–O layer. Even transmission of next-nearest-neighbour interactions proceeds within the magnetic layers via *M*–O–O–*M* super-superexchange pathways, as demonstrated for example in La_2CuO_4.[8] Our findings therefore suggest that additional exchange interactions involving rocksalt layers may also play a role in RP-materials.

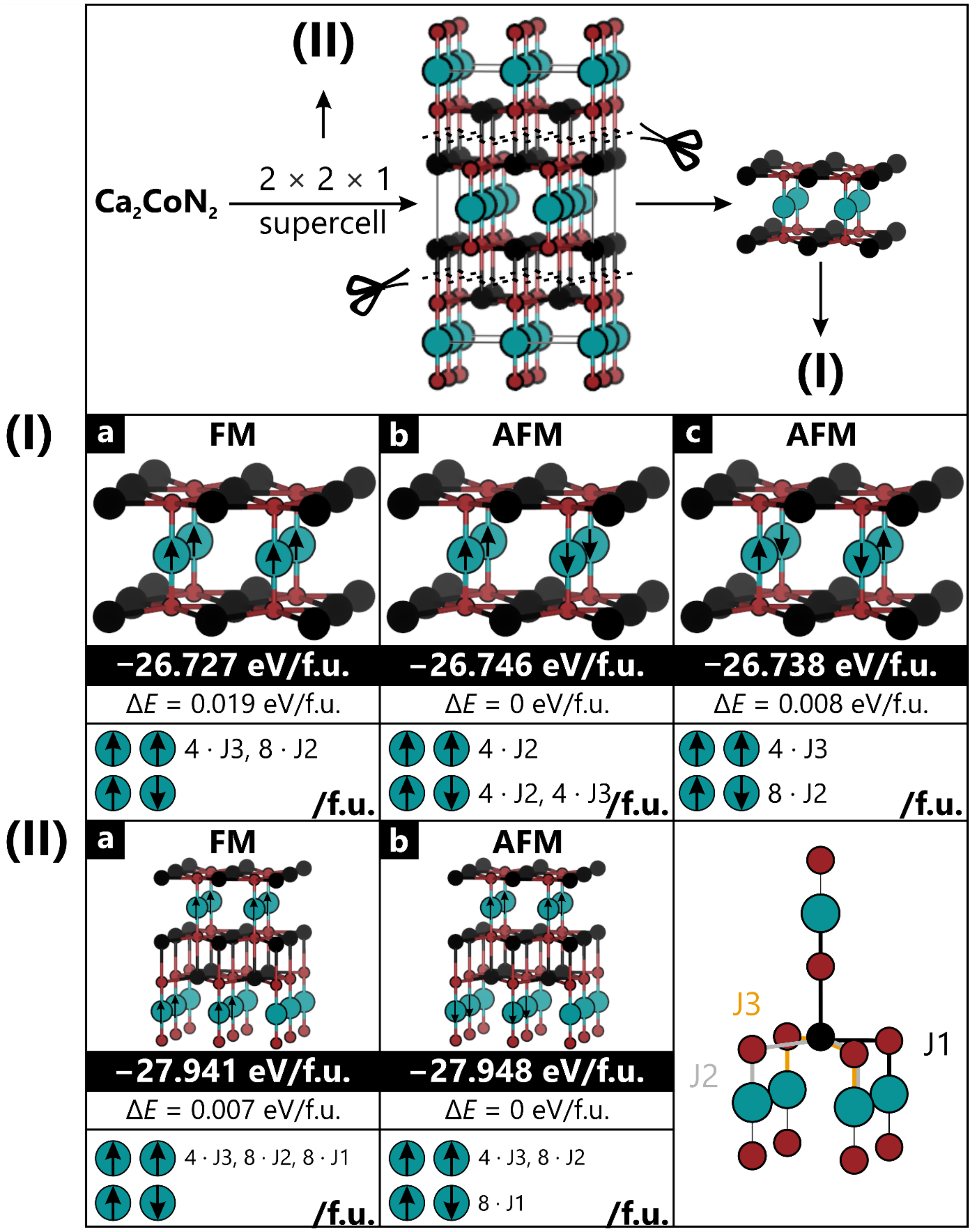

Supplementary Figure 5. Top: all models were derived from 2 × 2 × 1 expansions of the optimized structure model. In the case of set (II), the magnetic structures were simulated based on the 2 × 2 × 1 supercell, while one of the layers was removed in the models of the series (I). All total energies and corresponding spin configurations are provided per formula unit (f.u.), while the possible superexchange pathways are shown in the inset on the bottom left. Inset (I)a shows ferromagnetic interactions, (I)b and (I)b two different possibilities for antiferromagnetic arrangements in the layers. Inset (II)a shows ferromagnetic alignment between the layers, (II)b antiferromagnetic alignment.

Considerations on the magnetic exchange interactions in Ca_2CoN_2

DFT framework for calculating exchange interactions

The experimental data indicated a complex magnetic ground state stemming from the superexchange interactions between the spins localized on the cobalt ions. Therefore, the superexchange interactions are modelled with computational means in the following. First, a magnetic structure model that approximates the experimentally determined structure was constructed, where spin-orbit coupling[9] was also taken into consideration as a starting parameter of the spin-polarized DFT-based computations. These attempts, however, did not converge in the initial steps of the structural optimizations, as it was unfeasible to depict the complex spin states by means of the selected kind of functional. Therefore, a DFT+*U*-based approach,[10] which included an initial screening for an appropriate Hubbard *U* parameter[11] and was restricted to commensurate collinear magnetic starting models, was taken into account (see Computational Details section). A model that approaches the experimentally magnetic structure would require an individual *U* screening for each cobalt site and was omitted for technical reasons.

To evaluate the relative strength of the different superexchange pathways (inset on the bottom-right in Supplementary Figure 5) a series of superstructure models with certain magnetic orientations were calculated. To obtain the respective exchange constants J_{ij} from the results of DFT-based computations, it is required to determine the difference between the total energies corresponding to a ferromagnetic model and an antiferromagnetic one and to divide that amount of energy by the respective number of superexchange pathways.[12] In the framework of the Ca_2CoN_2 model, it is however not feasible to determine the total energies for each kind of plausible superexchange pathway independently. Nevertheless, the total energies for diverse models that correspond to 2 × 2 × 1 expansions of the optimized structure model were determined in order to estimate relative strengths of the exchange interactions. For accurate determinations of the total energies, the tetrahedron method[13] with Blöchl corrections and an enhanced **k**-points set of 20 × 20 × 12 for each calculation was used, while the supercells were generated by means of the phonopy[14,15] code.

Modelling the exchange interactions

As shown in Supplementary Figure 5, two different sets of Ca_2CoN_2 models based on 2 × 2 × 1 expansions have been investigated. Models (I)-a, -b, and -c are used for estimating the intralayer exchange while models (II)-a and (II)-b are used for the interlayer exchange. Model (I)-a has ferromagnetic alignment of all atoms, (I)-b has antiferromagnetic alignment in one direction and ferromagnetic in the other, while (I)-c is a checkerboard antiferromagnetic state. Model (II)-a has ferromagnetic alignment of layers while (II)-b has antiferromagnetic alignment of layers. All parameters discussed in the following are scaled to a formula unit (f.u.) for a better comparison. An analysis of both series shows that an antiferromagnetic configuration is clearly favoured in all cases being in good agreement with the experimentally determined results.

Intralayer Exchange

A comparison of the antiferromagnetic models (I)-b and -c reveals that the configuration (I)-b corresponds to the lowest total energy. This is in line with the observed experimental models showing frustration owing to competing ferromagnetic and antiferromagnetic interactions. A clear difference between both models can be recognized for the exchange constants J_2 and J_3. In (I)-c, all exchange

constants J_2 transmitting spin information via 90° angles are related to antiferromagnetic configurations, while the exchange parameters J_3 with 180° angles correspond to parallel alignment of the spins. In model (I)-b four of the J_2 constants are connected to a parallel configuration of the spins in, but all J_3 and four additional J_2 parameters are related to antiparallel spin arrangements the Ca_2CoN_2 model. A comparison of the total energies for models reveals that a scenario where all J_3 parameters are linked to parallel arrangements of the spins is energetically less favourable than a situation, which requests that half of the J_2 constants are linked to a parallel alignment of the spins. This outcome is in line with the Goodenough-Kanamori-Anderson rules which state that the exchange interactions are preferably AFM for 180° and FM for 90° angles. In this connection, it should also be noted that it is difficult to separate the different superexchange pathways, because replacing one of the pathways necessitates a change of another as can be recognized from the insets which summarize the respective spin configurations in relation to the different exchange pathways (see e.g. Figures S5 (I)-b and -c). As the transformations related to both superexchange pathways cause a certain change in energy, it is not possible to investigate the nature of the intralayer pathways independently. At this point, one may wonder whether it is possible to construct alternative models; however, such configurations would result in dissimilar compositions, i.e. Ca_2CoN_4, whose total energies cannot be compared to the other models in the framework of DFT-based computations.[16]

In summary, the comparison of the total energies reveals that there is a clear preference towards an AFM arrangement where an antiparallel configuration prevails for the superexchange pathways with a 180° angle. This outcome is in line with the Goodenough-Kanamori-Anderson rules and also means that the superexchange interactions corresponding to the constants J_3 provide a higher gain in energy and are stronger than the those related to J_2 ($J_2 < J_3$). This agrees with the orbital overlap being stronger for the linear combination relative to the angled one.[17] While our analysis for the series (I) could help us to gain an insight into the nature of the intralayer superexchange pathways, we will now continue with our investigation for the interlayer exchange interactions.

Interlayer Exchange

Models (II)-a and -b are used to analyse the interlayer superexchange interactions and the respective constant J_1. At first glance the total energies/f.u. of the models (II) are much higher than those of the models (I), however, in models (II) two layers are present, thus additional bonds that contribute to the net total energies.[18] The spins are aligned ferromagnetically within the layers of the models (II)-a and -b, while there are a ferromagnetic interlayer coupling in model (II)-a and an antiferromagnetic interlayer exchange model (II)-b. Accordingly, the energy difference between the models (II)-a and -b is supposed to primarily arise from the changes related of the superexchange parameters J_1. The total energy difference between both models of the set (II) (0.007 eV/f.u.) is even smaller than that between the models (I)-a and (I)-c (0.011 eV/f.u.), although the latter contains unfavourable parallel configurations for the superexchange constants J_3. Therefore, it can be concluded that the interlayer superexchange interactions are weaker than the intralayer one. As a result, the relative energies of the exchange interactions are $J_1 < J_2 < J_3$, which agrees with predictions on the bct-type lattice discussed in the main article.

Synchrotron X-ray Diffraction

Additional high-resolution synchrotron powder X-ray diffraction measurements were carried out at the ID22 beamline of the ESRF (Supplementary Figure 6 and 7, for tetragonal and orthorhombic model respectively) at 4 and 100 K. These measurements were carried out to investigate a possible orthorhombic lattice distortion during the magnetic phase transition. Pawley-fits to the measured data were carried out in the original tetragonal cell with space group *I*4/*mmm* (no. 139) and in its maximal *translationengleiche* subgroup *Immm* (no. 71). The Pawley-fit ensures no structural bias as reflections are modelled without structural information. The results of the refinements are shown in Table S5.

The *a* and *b* lattice parameters between the tetragonal and orthorhombic models are equal to the fourth decimal place. There, the *a* and *b* lattice parameters deviate for both the 100 and the 4 K measurement. However, the deviation is not significant as the refined *a* and *b* parameters are equal within 3σ. Moreover, the same deviation is observed for the 100 K and 4 K refinements and therefore reflects the added degree of freedom by introducing an additional parameter and not a real orthorhombic lattice. This in accordance with visual inspection of the weak reflections at high Q-values (Supplementary Figure 6b), where no peak splitting is observed. The accuracy of the unit cell parameters is of the order 10^{-5} Å in the tetragonal case, which should reveal even very small metric distortions. Therefore, Ca_2CoN_2 remains tetragonal below the magnetic transition, which is a good indication for the correctness of the double k magnetic structure.

Table S5: Lattice parameters, volume and R_{Bragg} for the tetragonal and orthorhombic models at 4 and 100 K.

	4 K – Tetragonal	4 K – Orthorhombic	100 K – Tetragonal	100 K – Orthorhombic
Lattice Parameters / Å	*a* = 3.56293(1)<br>*c* = 12.42102(6)	*a* = 3.5625(1)<br>*b* = 3.5630(1)<br>*c* = 12.42051(5)	*a* = 3.56440(1)<br>*c* = 12.42206(6)	*a* = 3.5640(1)<br>*b* = 3.5645(1)<br>*c* = 12.42051(5)
Volume / Å^3	157.678(1)	157.661(7)	157.821(1)	157.807(6)
R_{Bragg} / %	0.83	1.25	1.06	0.98
R_p, R_{wp} / %	8.6, 11.5	8.6, 11.5	9.1, 12,1	9.4, 12.2
GOF	6.0	6.0	7.5	7.6

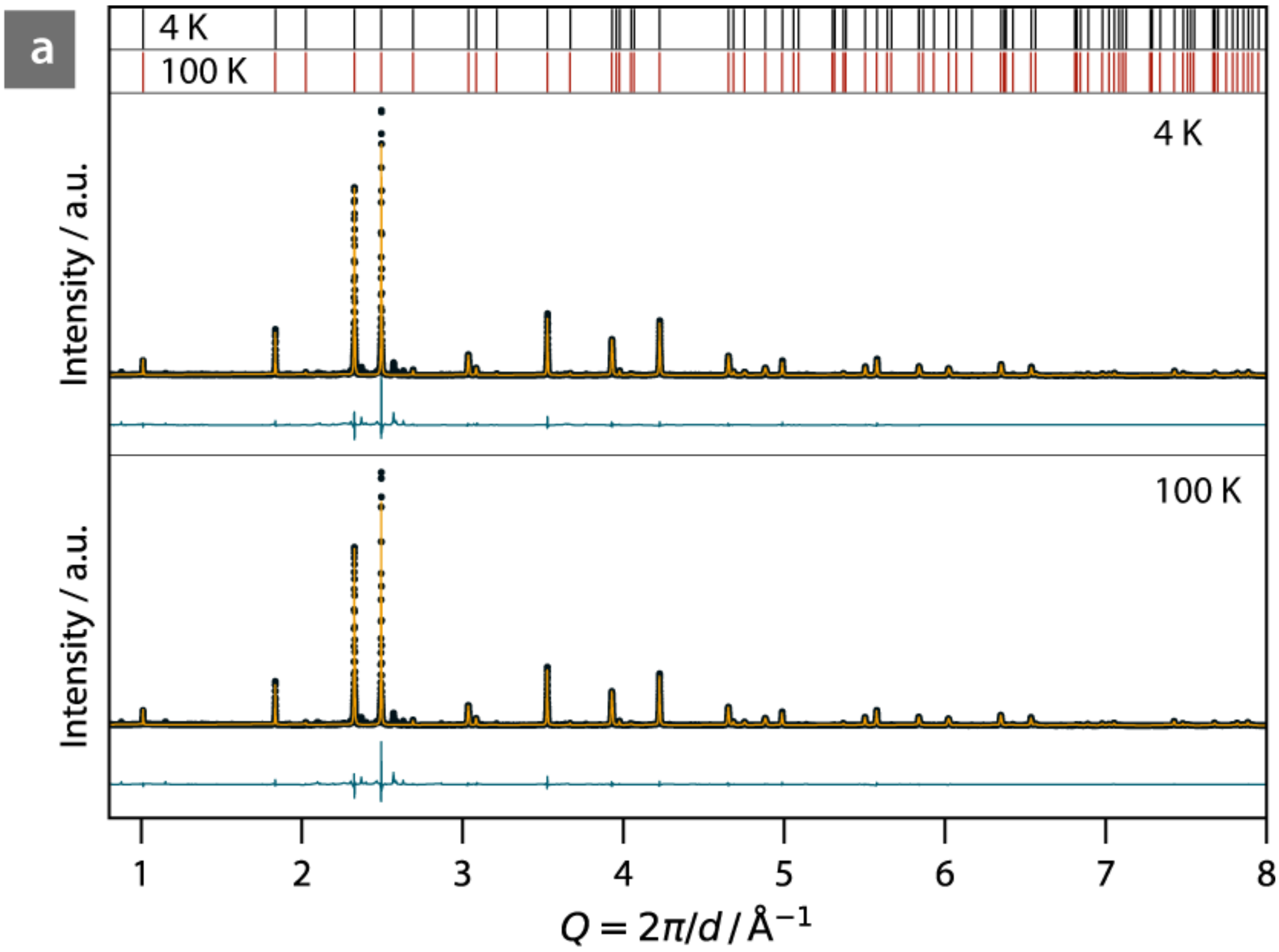

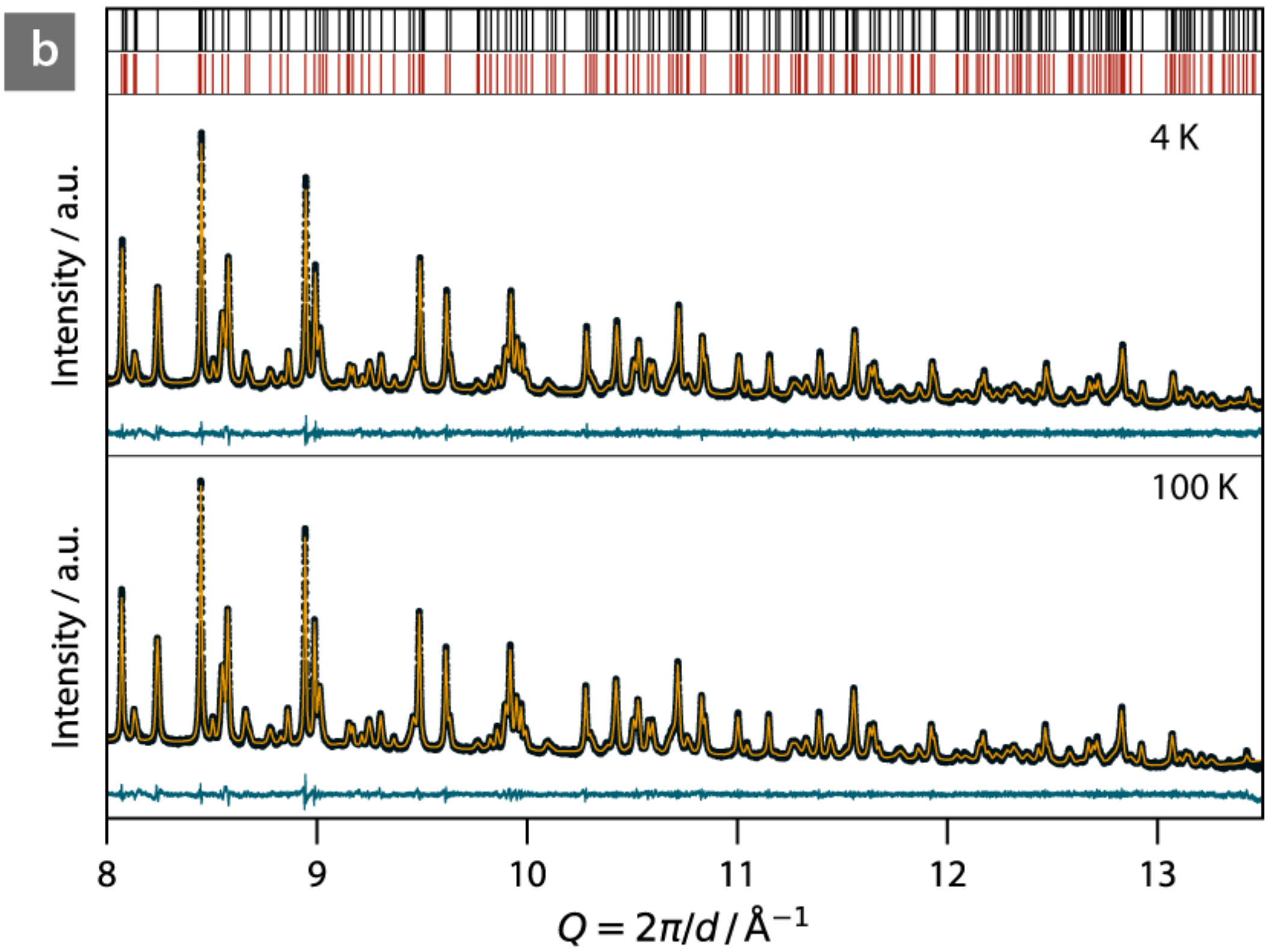

Supplementary Figure 6. Synchrotron powder X-ray diffraction on Ca_2CoN_2 collected at the ID22 - High resolution powder diffraction beamline of the ESRF at temperatures of 100 and 4 K. **a** Measured data in the Q-range from 0.8 to 8 $Å^{-1}$ and **b** in the range Q = 8–13.5 $Å^{-1}$. Measured data as black circles, Pawley-Fit in orange using the tetragonal model, difference curve in blue. Black tick marks indicate reflection positions at 4 K and red tick marks positions at 100 K.

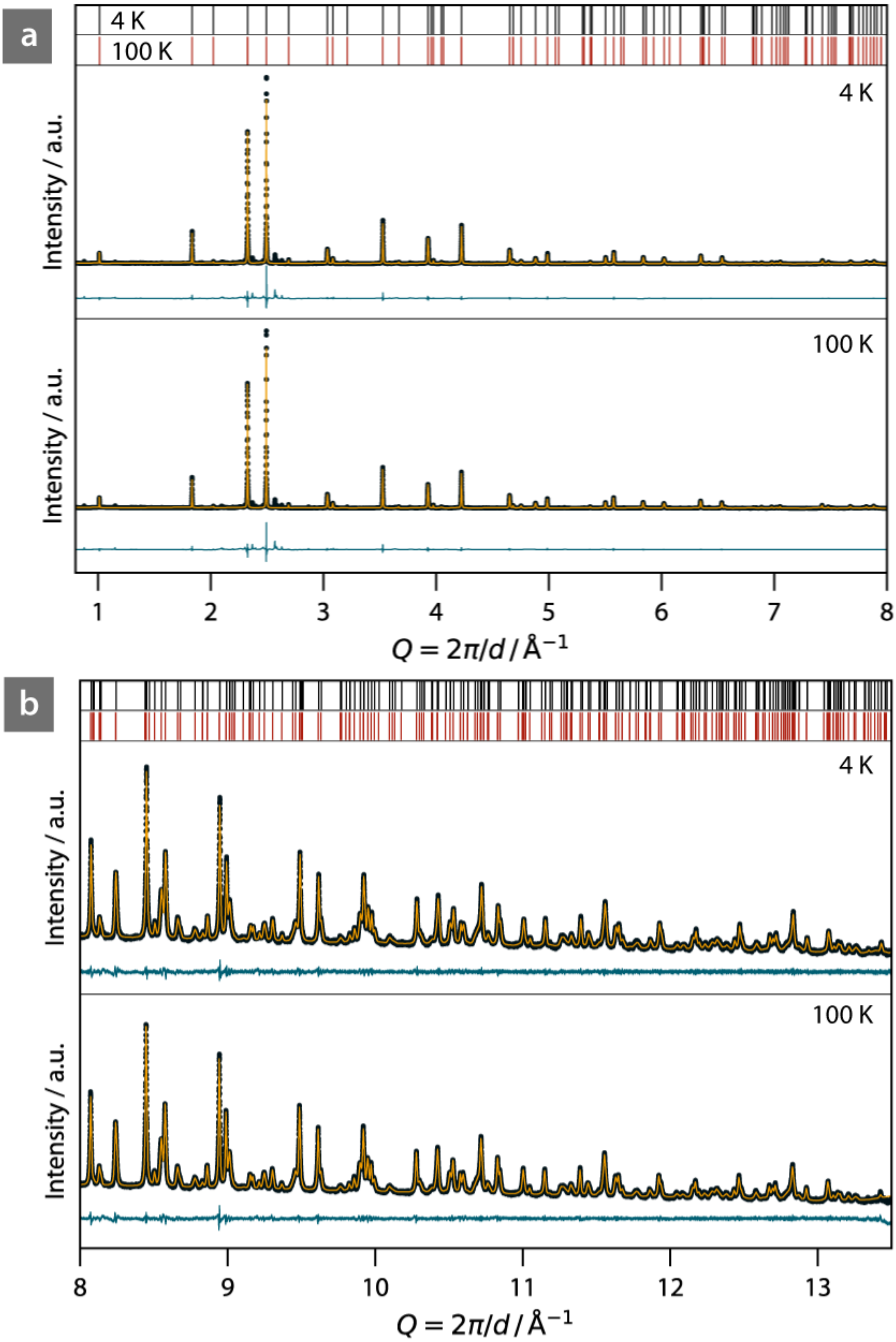

Supplementary Figure 7. Synchrotron powder X-ray diffraction on Ca_2CoN_2 collected at the ID22 - High resolution powder diffraction beamline of the ESRF at temperatures of 100 and 4 K. **a** Measured data in the Q-range from 0.8 to 8 $Å^{-1}$ and **b** in the range Q = 8–13.5 $Å^{-1}$. Measured data as black circles, Pawley-Fit in orange using the orthorhombic model, difference curve in blue. Black tick marks indicate reflection positions at 4 K and red tick marks positions at 100 K.

Additional Information on Magnetic Powder Neutron Diffraction

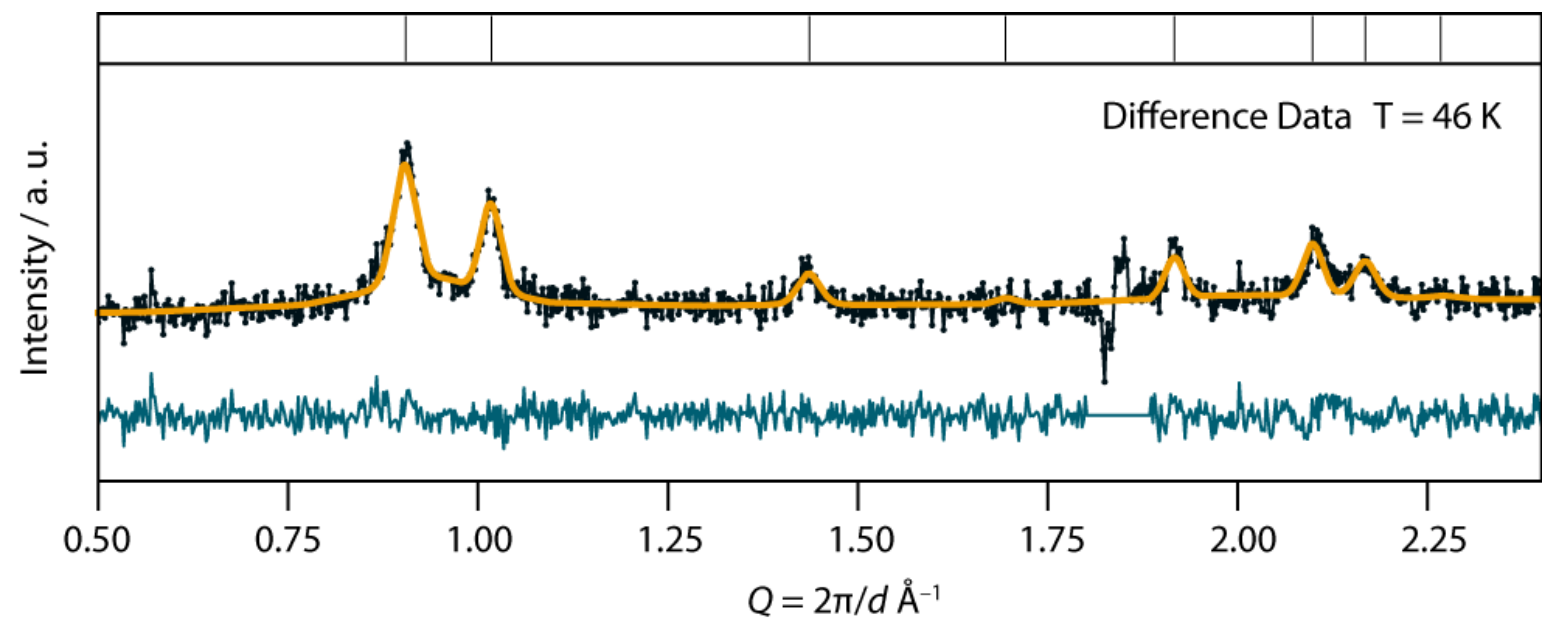

Supplementary Figure S8. Fit of the incommensurate magnetic structure to difference neutron diffraction data I_{46K}-I_{100K} with data in black, fit in orange, difference in blue. Reflection positions of the magnetic structure are shown as vertical black lines.

References

1 Rodriguez-Carvajal, J., Gonzalez-Platas, J. & Katcho, N. A. Magnetic structure determination and refinement using FullProf. *Acta Crystallogr B Struct Sci Cryst Eng Mater* **81**, 302-317 (2025). https://doi.org/10.1107/S2052520625003944

2 Campbell, B. J., Stokes, H. T., Tanner, D. E. & Hatch, D. M. ISODISPLACE: a web-based tool for exploring structural distortions. *J. Appl. Crystallogr.* **39**, 607-614 (2006). https://doi.org/10.1107/s0021889806014075

3 Reitz, L. S., Hempelmann, J., Müller, P. C., Dronskowski, R. & Steinberg, S. Bonding Analyses in the Broad Realm of Intermetallics: Understanding the Role of Chemical Bonding in the Design of Novel Materials. *Chem. Mater.* **36**, 6791-6804 (2024). https://doi.org/10.1021/acs.chemmater.4c00425

4 Hayami, S., Komatsu, Y., Shimizu, T., Kamihata, H. & Lee, Y. H. Spin-crossover in cobalt(II) compounds containing terpyridine and its derivatives. *Coord. Chem. Rev.* **255**, 1981-1990 (2011). https://doi.org/10.1016/j.ccr.2011.05.016

5 Atanasov, M. & Angelov, S. Angular overlap model parameterisation of Anderson's superexchange theory. I. A quantification of Goodenough-Kanamori rules. *Chem. Phys.* **150**, 383-393 (1991). https://doi.org/10.1016/0301-0104(91)87111-8

6 Shrestha, S. *et al.* Emergent interlayer magnetic order via strain-induced orthorhombic distortion in the 5d Mott insulator Sr2IrO4. *Phys. Rev. B* **105** (2022). https://doi.org/10.1103/PhysRevB.105.L100404

7 Meskine, H. & Satpathy, S. Electronic structure and magnetism in sodium nickelate: Density-functional and model studies. *Phys. Rev. B* **72** (2005). https://doi.org/10.1103/PhysRevB.72.224423

8 Coldea, R. *et al.* Spin waves and electronic interactions in La2CuO4. *Phys. Rev. Lett.* **86**, 5377-5380 (2001). https://doi.org/10.1103/PhysRevLett.86.5377

9 Steiner, S., Khmelevskyi, S., Marsmann, M. & Kresse, G. Calculation of the magnetic anisotropy with projected-augmented-wave methodology and the case study of disordered Fe1-xCox alloys. *Phys. Rev. B: Condens. Matter Mater. Phys* **93**, 224425 (2016). https://doi.org/10.1103/PhysRevB.93.224425

10 Anisimov, V. I., Zaanen, J. & Andersen, O. K. Band theory and Mott insulators: Hubbard *U* instead of Stoner *I*. *Phys. Rev. B: Condens. Matter Mater. Phys.* **44**, 943-954 (1991). https://doi.org/10.1103/PhysRevB.44.943

11 Rohrbach, A., Hafner, J. & Kresse, G. Electronic correlation effects in transition-metal sulfides. *J. Phys.: Condens. Matter* **15**, 979 (2003). https://doi.org/10.1088/0953-8984/15/6/325

12 Kresse, G., Marsman, M. & Furthmüller, J. *Vienna Ab-initio Simulation Package VASP: the Guide*. (2014).

13 Blöchl, P. E., Jepsen, O. & Andersen, O. K. Improved tetrahedron method for Brillouin-zone integrations. *Physical Review B: Condens. Matter Mater. Phys.* **49**, 16223-16233 (1994). https://doi.org/10.1103/PhysRevB.49.16223

14 Togo, A. First-principles phonon calculations with phonopy and phono3py. *J. Phys. Soc. Jpn.* **92**, 012001 (2023). https://doi.org/10.7566/JPSJ.92.012001

15 Togo, A., Chaput, L., Tadano, T. & Tanaka, I. Implementation strategies in phonopy and phono3py. *J. Phys. Condens. Matter* **35**, 353001 (2023). https://doi.org/10.1088/1361-648X/acd831

16 Gladisch, F. C. & Steinberg, S. Revealing Tendencies in the Electronic Structures of Polar Intermetallic Compounds. *Crystals* **8**, 80 (2018). https://doi.org/10.3390/cryst8020080

17 Albright, T. A., Burdett, J. K. & Whangbo, M.-H. *Orbital Interactions in Chemistry*. (John Wiley & Sons, 2013).

18 Miller, G. J. The "Coloring Problem" in Solids: How It Affects Structure, Composition and Properties. *Eur. J. Inorg. Chem.* **1998**, 523-536 (1998). https://doi.org/10.1002/(SICI)1099-0682(199805)1998:5<523::AID-EJIC523>3.0.CO;2-L
