# Supplementary material for: Complex magnetism in a transition metal nitride
Source: Nat Commun. 2026 Nov 2;17:11331. doi: 10.1038/s41467-026-78995-8 (PMC13728190; doi:10.1038/s41467-026-78995-8)
Supplement: Supplementary file 2 [file 41467_2026_78995_MOESM2_ESM.pdf]

nature portfolio

Peer Review File

Complex magnetism in a transition metal nitride

Corresponding Author: Dr Simon Kloss

This file contains all reviewer reports in order by version, followed by all author rebuttals in order by version.

Version 0:

Reviewer comments:

Reviewer #1

(Remarks to the Author)
In this paper, the authors present the synthesis and characterization of an n=1 Ruddlesden-Popper nitride $CaCo_2N_2$. The structure of this material is an anion-vacancy-ordered version of the K_2NiF_4 structure. Characterization of the magnetic properties reveals spin frustration originating from multiple superexchange pathways. The authors discover an incommensurate spin order, which can be described as a “double-k” spin density wave, that has previously been predicted by theory to be in close proximity to a spin liquid phase.

Overall I find the results in the paper to be of high quality and the presentation is generally clear. Synthesizing a new transition metal nitride hosting an exotic spin order is a significant research result which would be of interest to a broad scientific community and thus could be a good fit for Nature Communications. However, the following points should be addressed before considering acceptance.

In the Introduction, please improve the description of this work’s impact. Especially given that a similar group of authors already published on the synthesis of several other transition metal nitrides (Nature Chemistry, 2024), what is the significance of synthesizing $CaCo_2N_2$ compared to the compounds previously reported? Also, what in particular is interesting or the overall motivation for pursuing nitrides (beyond being new materials)? Are there any properties or potential applications for which transition metal nitrides are more promising than the more common oxides or halides?

The discussion of how the relative sizes of (J_1, J_2, J_3) relate to the stabilization of an incommensurate magnetic ground state (lines 217-221) is quite unclear, for example, it is not clear from the text why k = 0.57 implies the certain relation between J’s cited on line 220, and how that relation relates to the relation on line 219. Please improve the description and logic in this section.

I suggest that the authors estimate values for J_1, J_2, J_3 using DFT calculations. If the values follow the relation cited in line 219 for $CaCo_2N_2$, this would increase the evidence for the claimed incommensurate spin order.

I think the DFT calculations on collinear magnetic order, as reported in the text, are sufficient for supporting the authors’ points in this study, but it is a bit concerning that they report they tried DFT calculations on models approximating the incommensurate order (I assume this is the 7x7 superstructure shown in Fig. 4c?) and with spin-orbit coupling, but they failed to converge during structural relaxation. Did the authors try other approaches like pre-relaxing the structure without SOC, and only then turning on SOC/non-collinear calculations with the structure fixed? It would be useful if the authors could provide more detail on their attempts to calculate the magnetic superstructure using DFT. If possible to obtain, a converged superstructure calculation (and comparison of its total energy to the collinear case) would increase the evidence for the claimed magnetic structure in the paper.

The authors mention that small amounts of an unknown impurity are contained in the samples (line 71). Could this impact the magnetic properties? How confident are the authors that the observed behaviors are properties of the intrinsic sample?

Reviewer #2

(Remarks to the Author)
I co-reviewed this manuscript with one of the reviewers who provided the listed reports. This is part of the Nature

Communications initiative to facilitate training in peer review and to provide appropriate recognition for Early Career Researchers who co-review manuscripts.

Reviewer #3

(Remarks to the Author)
In this work, the authors report Ca_2CoN_2 as a rare example of a transition metal nitride with a Na_2HgO_2 type structure and frustrated magnetic interactions that lead to an unusual incommensurate magnetic order below 54 K. The magnetic structure is described as a complex spin density wave state, which is theoretically predicted to lie near a spin liquid phase.

Considering the novelty of the material synthesized under extreme conditions, as well as the rare observation of an incommensurate ordered state suggesting strong competition between nearest and next nearest neighbor couplings in the bct lattice, I consider the results to meet the journal's criteria for novelty and scientific advancement. However, several aspects of the discussion and interpretation require significant revision before the paper can be considered for publication. Below I list the points that need to be addressed:

I believe the authors should include a more thorough discussion of the origin of the amplitude modulated spin density wave (SDW) ground state and make it clear that the incommensurability is a consequence of competing exchange interactions, while the strong Co^{2+} easy axis anisotropy enforces the collinearity of the transverse SDW. It would also be beneficial to discuss other Co systems where similar SDW magnetic order has been observed.

My main concern relates to the authors' argument that the double-k model is more justified than a single-k model. As the authors correctly mentioned, the powder data cannot distinguish between the two possibilities. They state that "a single k structure entails ferromagnetic alignment of spins in one direction within the ab plane, which is unphysical in a tetragonal lattice without a structural distortion." I would argue that, in the present case, the strong single-ion anisotropy constrains the spins to align along the c axis, reducing the number of magnetic degrees of freedom. A collinear single-k structure therefore provides the simplest description of the observed data without requiring symmetry restoring combinations of equivalent propagation vectors. In fact, a double-k structure is generally less favored than a single-k structure unless higher order coupling terms explicitly stabilize it. Without additional support from single crystal studies, the double-k solution remains speculative.

Another aspect that should be addressed is the interpretation that an amplitude modulated SDW implies that a significant fraction of spins remain paramagnetic. In a long range SDW, all spins participate in the collective ordered state, and the local order parameter amplitude varies in space. The spins remain strongly correlated and do not fluctuate independently in a way that would produce Curie–Weiss behavior.

I also disagree with the explanation attributing the gradual temperature variation of the k vector, from 0.566 to about 0.58, to thermal expansion of the lattice. Such temperature dependence is quite typical for incommensurate SDWs and often corresponds to changes in the balance between short range correlations and anisotropy terms, which can shift the minimum of the magnetic free energy.

I would also recommend that the authors adopt the same notation for J_1-J_2-J_3 as used in Reference 32 (Farias, C. et al, 2016). A completely different labeling, as used in the current version, could easily lead to confusion unless clearly explained.

More information is also needed regarding the hysteresis loop shown in Supplementary Figure 3. The authors attribute this behavior to an impurity, but it would be helpful to explain this conclusion more clearly. Data just above the ordering temperature would be useful for comparison. A weak ferromagnetic component arising from spin canting can produce a magnetization curve that looks similar to that of a small ferromagnetic impurity, especially in powder samples.

It seems that the authors used the ISODISTORT web based tool to obtain the magnetic superspace group (I4/mmm.1'(a,0,0)0000s(0,a,0)00s0s)) , but the manuscript does not properly reference or acknowledge this tool. Additionally, the authors should provide the corresponding superspace group information for the single-k model as well.

While I appreciate that the authors provided the magnetic-CIF files for the magnetic models, these files should be clearly referenced and described in the supplementary information.

Finally, I note that I am not an expert in DFT calculations and therefore refrain from evaluating that part of the manuscript.

To summarize, the study is interesting and has real potential, but the magnetic analysis needs substantial clarification and revision before the manuscript can be considered for publication.

Reviewer #4

(Remarks to the Author)
The authors have investigated the magnetic order in Ca_2CoN_2 synthesized under high pressure by means of neutron diffraction, magnetization measurements and first-principles calculations. They have refined the crystal structure by neutron powder diffraction measurements. They also performed magnetization measurements, revealing an antiferromagnetic

transition at TN=62 K. The magnetization shows a kink anomaly at TN, but it is further increased as the temperature is decreased, indicating the existence of paramagnetic moments even below TN. They performed Rietveld analysis on the observed neutron powder diffraction patterns, and concluded that the magnetic ground state is a double-q incommensurate magnetic phase. They also performed DFT calculations to estimate the exchange interactions between the magnetic moments on the Co sites. The results of the calculations qualitatively agree with the arrangements of the magnetic moments deduced from the experiments.
These measurements were properly done. The quality of the data shown in the manuscript is relatively high. However, in my opinion, this manuscript does not meet the criteria for publication in Nat. Commun. as described below.

(1) The main issues of this manuscript are the synthesis of the nitrogen-based magnet and the proposal of double-q magnetic structure. As the authors mentioned in the main text, A2BN2-type nitrometallates are relatively rare. However, there are a number of previous studies reporting the synthesis of nitromatallates as listed in the references 16, 19-22. If the authors put a stress on the synthesis of this particular compound, this manuscript should be submitted to more material-oriented journals.

(2) The proposed magnetic structure seems to have only the c-axis component of the magnetic moments. Thus, the incommensurate magnetic order is not a helical magnetic order but a sinusoidally modulated structure. Although the authors mentioned the similarity between this double-q structure and magnetic skyrmions, this double-q sinusoidal structure is topologically trivial. It would be difficult to expect this study to have an impact in the research field of magnetic skyrmions and related topological magnetic orders. Even if the double-q picture is correct, the impact of this research is not enough to be published in Nat. Commun.

(3) In this manuscript, the double-q magnetic order scenario is supported mainly by the fact that the authors did not observe any obvious splitting or broadening of nuclear reflections observed by their neutron powder diffraction measurements. In general, the symmetry-lowering lattice distortions associated with single-q magnetic orders are very small, even in 3d-transition-metal oxides. For instance in multiferroic CuCrO2, the magnetostriction associated with the formation of the single-q order was reported to be dL/L~10^-4 (J. Phys. Soc. Jpn. 78, 113710 (2009)). The authors should provide the resolution limit of the neutron powder diffraction measurements and lower limit of the detectable lattice distortion. Since the formation of the double-q order relies on the assumption that there is no lattice distortion, the proposed picture of the magnetic structure is not convincing enough. Normally, to confirm the formation of the multi-q states, measurements of higher-order magnetic reflections indexed by q1+q2 (=(q,0,0)+(0,q,0)=(q,q,0)) are carried out (for instance Nat. Nanotech. 15, 444 (2020).)

(4) It should also be pointed out that the sinusoidal magnetic structures contain the magnetic sites whose magnetic moments are disordered, regardless of the number of q-vectors. The authors ascribed the upturn of the magnetic susceptibility below TN to the partially paramagnetic moments. This kind of partial-orders often appear in frustrated magnetic systems at finite temperatures. As the temperature is decreased the fluctuating magnetic moments tend to be frozen, and the magnetic modulation would be squared up, which often results in higher-harmonic peaks indexed as 2q, 3q… Although this kind of higher harmonic peaks are expected to be weak, the authors should mention whether they were observed or not.

Version 1:

Reviewer comments:

Reviewer #1

(Remarks to the Author)
I have reviewed the revised manuscript. I find that the authors largely addressed my concerns from the previous report, thus I recommend publication assuming that the authors has answered the concerns from the other referees in a satisfactory manner.
However, I do not want mention that some of the authors' discussion of the DFT calculations seems a bit unclear and of questionable validity. For example, in the reply letter they say "a proper description of the respective experimentally observed magnetic moments requests to treat the functional of each cobalt atom independently. As correlation and exchange cannot be properly described by the available sorts of functionals, an individual Hubbard U parameter must be included for every cobalt site. Accordingly, such a procedure also means that a Hubbard U screening has to be accomplished for each cobalt site within the supercell. Unfortunately, such computations represent a problem to the architecture of the used VASP code."

I don't think that one can treat the "functional of each cobalt atom independently" within a standard DFT code, to my knowledge. One would choose a functional (e.g. PBE or LDA) and use that choice for all elements in the calculation. I'm guessing that the authors are saying that they are using DFT+U to account for the on-site correlation on Co, which is correct. However, this is a fairly standardized approach, where one sets U on each magnetic site (in this case Co), so it is not clear to me why this would pose a problem "to the architecture of the vasp code." Since the primary novelty of the paper is based on the experimental results, I don't think these issues should hinder the paper from moving forward. On the other hand, it would be useful if the authors could clarify what they mean by their statements regarding DFT and its limitations for their material, in order to improve confidence that the calculations were conducted in the appropriate way.

Reviewer #2

(Remarks to the Author)
I co-reviewed this manuscript with one of the reviewers who provided the listed reports. This is part of the Nature Communications initiative to facilitate training in peer review and to provide appropriate recognition for Early Career Researchers who co-review manuscripts.

Reviewer #3

(Remarks to the Author)
The authors have improved the manuscript and clarified the points raised in my previous report. They have also provided additional support for their main claim regarding the possibility of a double-k magnetic structure. In my view, either a single-k or a double-k magnetic state would make this system interesting, and presenting the details of both models provides a clear assessment of the possible interpretations consistent with the available data. Considering the novelty of Ca2CoN2 and its unusual incommensurate magnetic state, I believe that the manuscript is of sufficient interest and significance and is now in good shape for publication.

Reviewer #4

(Remarks to the Author)
The authors have adequately addressed the issues raised in the previous review round, and revised the manuscript accordingly. In particular, the additional high-resolution XRD measurements using synchrotron radiations source and the assignment of the (q,q,0) reflection are very valuable. The double-q magnetic structure is now plausible, and the discovery of the multi-q magnetic order in nitromatallates would attract attention in this research field. The revised manuscript would deserve to be published in Nat. Commun.

Open Access This Peer Review File is licensed under a Creative Commons Attribution 4.0 International License, which permits use, sharing, adaptation, distribution and reproduction in any medium or format, as long as you give appropriate credit to the original author(s) and the source, provide a link to the Creative Commons license, and indicate if changes were made.
In cases where reviewers are anonymous, credit should be given to 'Anonymous Referee' and the source.
The images or other third party material in this Peer Review File are included in the article's Creative Commons license, unless indicated otherwise in a credit line to the material. If material is not included in the article's Creative Commons license and your intended use is not permitted by statutory regulation or exceeds the permitted use, you will need to obtain permission directly from the copyright holder.
To view a copy of this license, visit https://creativecommons.org/licenses/by/4.0/

Point-by-Point Response Letter

Manuscript Title: Complex magnetism in a transition metal nitride
Manuscript ID: NCOMMS-26-032407

REVIEWER COMMENTS

Reviewer #1 (Remarks to the Author):

In this paper, the authors present the synthesis and characterization of an n=1 Ruddlesden-Popper nitride $CaCo_2N_2$. The structure of this material is an anion-vacancy-ordered version of the K_2NiF_4 structure. Characterization of the magnetic properties reveals spin frustration originating from multiple superexchange pathways. The authors discover an incommensurate spin order, which can be described as a “double-k” spin density wave, that has previously been predicted by theory to be in close proximity to a spin liquid phase. Overall I find the results in the paper to be of high quality and the presentation is generally clear. Synthesizing a new transition metal nitride hosting an exotic spin order is a significant research result which would be of interest to a broad scientific community and thus could be a good fit for Nature Communications. However, the following points should be addressed before considering acceptance.

Response: Thank you for evaluating our manuscript.

In the Introduction, please improve the description of this work’s impact. Especially given that a similar group of authors already published on the synthesis of several other transition metal nitrides (Nature Chemistry, 2024), what is the significance of synthesizing $CaCo_2N_2$ compared to the compounds previously reported? Also, what in particular is interesting or the overall motivation for pursuing nitrides (beyond being new materials)? Are there any properties or potential applications for which transition metal nitrides are more promising than the more common oxides or halides?

Response: In magnetism in general, nitrides play an important role demonstrated by much studied materials classes such as permanent magnets such as $Fe_{16}N_2$ or nitromag $Sm_2Fe_{17}N_3$ and antiferromagnetic spintronic materials such as antiperovskite-type Mn-based nitrides. We added a discussion of the significance to the main article. Nitride offers larger covalency than oxides and halides, which strengthens superexchange interactions, which in principle is interesting for achieving higher ordering temperatures. Inasmuch, the synthesis of Ca_2CoN_2 is interesting as transition metal nitrides are generally more difficult to prepare; this is the first instance of a Co^{2+} nitridometallate, therefore discussion of synthesis of nitrides is more commonplace than with oxides. We agree, however, that the focus of this work is not the synthesis method that was reported before but the physical properties. These give significant insight into weak superexchange interactions in the important class of Ruddlesden-Popper materials as well as demonstrate that our material is a possible candidate to study the spin-liquid transition in a bct lattice. Therefore, our results are of direct interest to large research communities.

The discussion of how the relative sizes of (J1, J2, J3) relate to the stabilization of an incommensurate magnetic ground state (lines 217-221) is quite unclear, for example, it is not clear from the text why k = 0.57 implies the certain relation between J’s cited on line 220, and how that relation relates to the relation on line 219. Please improve the description and logic in this section.

Response: We thank the reviewer for pointing out this lack of clarity. The original text conflated two distinct conditions without explaining the logic connecting them. We have revised this section as follows.

The condition $J3 > 0.5 \cdot J2 > 0.25 \cdot J1$ is a stability condition taken from the phase diagram of ref. Farias et al., PRB 94, 134420, 2016. It defines the region of exchange parameter space, in which the incommensurate (k00)/(0k0) spiral is the lowest-energy ground state, as opposed to commensurate antiferromagnetic or other helical phases.

The relation $(J2 + 2J3) = 4.6 \cdot J1$ is a separate, independent result that follows from the observed value of k. Within the (k00) phase, the incommensurate wave vector is determined by minimising the exchange energy of a classical spin spiral over all possible ordering wave vectors. Inserting the experimentally observed k = 0.57 directly yields $(J2 + 2J3)/J1 = -1/\cos(\pi\, 0.57) \approx 4.6$. This is not an additional assumption but a consequence of k alone.

The two conditions therefore operate at different levels: the stability condition places Ca_2CoN_2 in the correct region of the Farias et al. phase diagram, while the wave vector condition extracts quantitative information about the exchange ratio from the observed k. We have rewritten lines 217–221 to make this distinction explicit.

I suggest that the authors estimate values for J1, J2, J3 using DFT calculations. If the values follow the relation cited in line 219 for CaCo2N2, this would increase the evidence for the claimed incommensurate spin order. I think the DFT calculations on collinear magnetic order, as reported in the text, are sufficient for supporting the authors' points in this study, but it is a bit concerning that they report they tried DFT calculations on models approximating the incommensurate order (I assume this is the 7x7 superstructure shown in Fig. 4c?) and with spin-orbit coupling, but they failed to converge during structural relaxation. Did the authors try other approaches like pre-relaxing the structure without SOC, and only then turning on SOC/non-collinear calculations with the structure fixed? It would be useful if the authors could provide more detail on their attempts to calculate the magnetic superstructure using DFT. If possible to obtain, a converged superstructure calculation (and comparison of its total energy to the collinear case) would increase the evidence for the claimed magnetic structure in the paper.

Response: Thank you for this point. Indeed, we have tried to accomplish spin-polarized computations for the superstructure as mentioned by the reviewer; however, a proper description of the respective experimentally observed magnetic moments requests to treat the functional of each cobalt atom independently. As correlation and exchange cannot be properly described by the available sorts of functionals, an individual Hubbard U parameter must be included for every cobalt site. Accordingly, such a procedure also means that a Hubbard U screening has to be accomplished for each cobalt site within the supercell. Unfortunately, such computations represent a problem to the architecture of the used VASP code. Also, that issue concerning the description of correlation and exchange for the cobalt sites could not be solved under consideration of spin-orbit coupling. Nevertheless, it is necessary to take the magnetism of Ca2CoN2 into account for the structural optimizations, because the atomic radius of the cobalt atoms strongly depends on the respective electronic configuration, e.g. high-spin vs. low-spin. Eventually, it was possible to reproduce the structure model and average magnetic moment by using the approach as described in the Computational Details section. We agree that this line of thought was not entirely clear in the original version of our submission, and a revised version describing our computational approach has been included as part of our considerations concerning the superexchange interactions in the Supporting Information.

Also, we agree with the reviewer that it will be quite helpful to estimate J1, J2 and J3 in order to support the analysis of the experimental data. However, it is problematic to construct models, which allow us to analyze the superexchange pathways independently, because the construction of such models would request to change the composition. Such a modification of the composition means that a direct comparison between the total energies of the diverse models cannot be made anymore, although the total energies must be compared in order to access the strength of the respective superexchange parameters.

We, however, have investigated another series of Ca_2CoN_2 models, which were constructed based on superstructures in order to project the strength of the different superexchange pathways. The comparison of these models suggests that that the relative exchange interaction strengths are the same as predicted for the bct model; 180°-type J3 > 90°-type J2 > interlayer J1. These calculations therefore further support the findings of our experimental investigations. We added our considerations of the exchange pathways, which include a detailed description concerning the development and analysis of the different models, to the revised version of the main Manuscript as well as the Supplementary Information.

The authors mention that small amounts of an unknown impurity are contained in the samples (line 71). Could this impact the magnetic properties? How confident are the authors that the observed behaviors are properties of the intrinsic sample?

Response: The impurities observed in the powder pattern are on the 5 % level from estimation of relative peak intensities (added to the manuscript). The magnitude of the observed magnetic transition in the susceptibility measurements as well as the powder neutron diffraction data indicate that these stem from the main phase. A Neél Transition of a minor byproduct, while the main phase remains paramagnetic would produce a weaker transition.

Reviewer #2 (Remarks to the Author):
I co-reviewed this manuscript with one of the reviewers who provided the listed reports. This is part of the Nature Communications initiative to facilitate training in peer review and to provide appropriate recognition for Early Career Researchers who co-review manuscripts.

Response: Thank you for evaluating our manuscript.

Reviewer #3 (Remarks to the Author):
In this work, the authors report Ca2CoN2 as a rare example of a transition metal nitride with a Na2HgO2 type structure and frustrated magnetic interactions that lead to an unusual incommensurate magnetic order below 54 K.

The magnetic structure is described as a complex spin density wave state, which is theoretically predicted to lie near a spin liquid phase.

Considering the novelty of the material synthesized under extreme conditions, as well as the rare observation of an incommensurate ordered state suggesting strong competition between nearest and next nearest neighbour couplings in the bct lattice, I consider the results to meet the journal's criteria for novelty and scientific advancement. However, several aspects of the discussion and interpretation require significant revision before the paper can be considered for publication.

Response: Thank you for evaluating our manuscript.

Below I list the points that need to be addressed:
I believe the authors should include a more thorough discussion of the origin of the amplitude modulated spin density wave (SDW) ground state and make it clear that the incommensurability is a consequence of competing exchange interactions, while the strong Co^{2+} easy axis anisotropy enforces the collinearity of the transverse SDW. It would also be beneficial to discuss other Co systems where similar SDW magnetic order has been observed.

Response: Thank you for pointing out this oversight. We added additional discussion to the powder neutron diffraction section as well as the conclusion. We compare our system with the Co-based oxide $Ca_3Co_2O_6$, where a similar amplitude modulated SDW was observed.

My main concern relates to the authors' argument that the double-k model is more justified than a single-k model. As the authors correctly mentioned, the powder data cannot distinguish between the two possibilities. They state that "a single k structure entails ferromagnetic alignment of spins in one direction within the ab plane, which is unphysical in a tetragonal lattice without a structural distortion." I would argue that, in the present case, the strong single-ion anisotropy constrains the spins to align along the c axis, reducing the number of magnetic degrees of freedom. A collinear single-k structure therefore provides the simplest description of the observed data without requiring symmetry restoring combinations of equivalent propagation vectors. In fact, a double-k structure is generally less favored than a single-k structure unless higher order coupling terms explicitly stabilize it. Without additional support from single crystal studies, the double-k solution remains speculative.

Response: We carefully re-evaluated our powder neutron diffraction data and investigated the observation of k_1+k_2 superstructure reflections. We do indeed observe weak magnetic intensity of the (110)-(kk0) superstructure reflection. Furthermore, we collected high-resolution synchrotron powder diffraction data at the ID22 beamline of the ESRF to check for metric distortions during the magnetic phase transition. Fits to these data show that the unit cell remains tetragonal down to 4K to the highest available resolution. Both of these points give good evidence for the correctness of the double k magnetic structure model. We agree that additional probes such as Muon spin relaxation may be helpful, but these experiments are outside the scope of this report and could be incorporated in future studies. We added an additional discussion the neutron diffraction section.

Another aspect that should be addressed is the interpretation that an amplitude modulated SDW implies that a significant fraction of spins remain paramagnetic. In a long range SDW, all spins participate in the collective ordered state, and the local order parameter amplitude varies in space. The spins remain strongly correlated and do not fluctuate independently in a way that would produce Curie–Weiss behavior.

Response: Thank you for pointing out this ambiguity in our explanation. We concur with the assessment if the system was an itinerant electron SDW as there the SDW state is a consequence of band structure nesting and the resulting ordered state is a reflection of the Fermi Surface reconstruction. However, in localized spin systems as it is the case here, spins on nodes of the SDW are free to align with the field leading to a paramagnetic-like response; this was for example shown for TmB_4 (Origin of modulated phases and magnetic hysteresis in TmB_4: https://www.osti.gov/pages/servlets/purl/1239160)

I also disagree with the explanation attributing the gradual temperature variation of the k vector, from 0.566 to about 0.58, to thermal expansion of the lattice. Such temperature dependence is quite typical for incommensurate SDWs and often corresponds to changes in the balance between short range correlations and anisotropy terms, which can shift the minimum of the magnetic free energy.

Response: Thank you for pointing out the ambiguity in our description; we changed the corresponding explanation.

I would also recommend that the authors adopt the same notation for J1-J2-J3 as used in Reference 32 (Farias, C. et al, 2016). A completely different labeling, as used in the current version, could easily lead to confusion unless clearly explained.

Response: We changed the labelling so that it is in line with Ref 32 Farias et al.

More information is also needed regarding the hysteresis loop shown in Supplementary Figure 3. The authors attribute this behavior to an impurity, but it would be helpful to explain this conclusion more clearly. Data just above the ordering temperature would be useful for comparison. A weak ferromagnetic component arising from spin canting can produce a magnetization curve that looks similar to that of a small ferromagnetic impurity, especially in powder samples.

Response: The impurity is probably from small amounts of Co-metal (remnants of the starting material), as there is a change in slope of the magnetisation measurement obtained at 300K as well. Therefore, the magnetic phase needs to have a T_C above room temperature. We clarified this in the figure caption.

It seems that the authors used the ISODISTORT web based tool to obtain the magnetic superspace group (I4/mmm.1'(a,0,0)0000s(0,a,0)00s0s)) , but the manuscript does not properly reference or acknowledge this tool. Additionally, the authors should provide the corresponding superspace group information for the single-k model as well.

Response: Thank you for pointing out this oversight. We added the corresponding references to the main manuscript as well as Supplementary Information. We also stated the superspace group for the single-k superstructure in the main manuscript as it was only reported in the Supplementary Information before.

While I appreciate that the authors provided the magnetic-CIF files for the magnetic models, these files should be clearly referenced and described in the supplementary information.

Response: Thank you for pointing out this oversight. We updated the Data Availability Statement indicating that the mCIF files are available for download. We additionally added further information to the Supplementary Information regarding the mCIF files. We further added the mCIFs for the incommensurate single k structure as well as the commensurate approximation as a 7x supercell.

Finally, I note that I am not an expert in DFT calculations and therefore refrain from evaluating that part of the manuscript.

To summarize, the study is interesting and has real potential, but the magnetic analysis needs substantial clarification and revision before the manuscript can be considered for publication.

Response: Thank you for your comments on our article.

Reviewer #4 (Remarks to the Author):
The authors have investigated the magnetic order in Ca2CoN2 synthesized under high pressure by means of neutron diffraction, magnetization measurements and first-principles calculations. They have refined the crystal structure by neutron powder diffraction measurements. They also performed magnetization measurements, revealing an antiferromagnetic transition at TN=62 K. The magnetization shows a kink anomaly at TN, but it is further increased as the temperature is decreased, indicating the existence of paramagnetic moments even below TN. They performed Rietveld analysis on the observed neutron powder diffraction patterns, and concluded that the magnetic ground state is a double-q incommensurate magnetic phase. They also performed DFT calculations to estimate the exchange interactions between the magnetic moments on the Co sites. The results of the calculations qualitatively agree with the arrangements of the magnetic moments deduced from the experiments. These measurements were properly done. The quality of the data shown in the manuscript is relatively high. However, in my opinion, this manuscript does not meet the criteria for publication in Nat. Commun. as described below.

Response: Thank you for evaluating our manuscript.

(1) The main issues of this manuscript are the synthesis of the nitrogen-based magnet and the proposal of double-q magnetic structure. As the authors mentioned in the main text, A2BN2-type nitrometallates are relatively rare. However, there are a number of previous studies reporting the synthesis of nitromatallates as listed in the references 16, 19-22. If the authors put a stress on the synthesis of this particular compound, this manuscript should be submitted to more material-oriented journals.

Response: Same answer as for Reviewer #1. The stress of this article is not the synthesis, as pointed out correctly, that it was published before. The preparation of transition metal nitrides is, however, not as well explored as for transition metal oxides, this is what makes the whole chemistry exciting at the same time. This is also the reason why synthesis is usually shortly discussed in articles dealing with new transition metal nitrides. The main point of our article is that the material presented shows fascinating physical properties; an incommensurate modulated spin density wave ground state on a bct-type lattice, with a wavevector that indicates close approximation to a spin liquid transition predicted by theory. Moreover giving unprecedented insight into the weak exchange interactions responsible for three dimensional magnetic ordering in the famous Ruddlesden-Popper-type materials highlighting that superexchange interaction mediated within rocksalt-type layers are significant. The material Ca2CoN2 is a representative of a whole class of anion-defect ordered RP-type phases that exist in the nitride, oxide and carbide space, all of which received limited attention, a fact that our work will likely change as these types of materials are of interest to chemists, materials scientists, and physicists alike. We updated the introduction to better highlight the significance of our work.

> (2) The proposed magnetic structure seems to have only the c-axis component of the magnetic moments. Thus, the incommensurate magnetic order is not a helical magnetic order but a sinusoidally modulated structure. Although the authors mentioned the similarity between this double-q structure and magnetic skyrmions, this double-q sinusoidal structure is topologically trivial. It would be difficult to expect this study to have an impact in the research field of magnetic skyrmions and related topological magnetic orders. Even if the double-q picture is correct, the impact of this research is not enough to be published in Nat. Commun.

Response: Similar argument as above. We noticed that the size and shape of the resulting magnetic structure is reminiscent of magnetic skyrmions but we do not claim that we made one or that this is a skyrmion. We have rewritten the parts where we make this analogy to remove any ambiguities. Therefore, it is unfair to measure the impact of this article in a field where it is not based. The impact of our paper is in demonstrating that complex magnetism can arise in localised-electron transition metal nitrides which are a new class of materials in the field. This work shows that topological magnetism is feasible in nitrides, even if the current spin structure does not have topological properties.

> (3) In this manuscript, the double-q magnetic order scenario is supported mainly by the fact that the authors did not observe any obvious splitting or broadening of nuclear reflections observed by their neutron powder diffraction measurements. In general, the symmetry-lowering lattice distortions associated with single-q magnetic orders are very small, even in 3d-transition-metal oxides. For instance in multiferroic CuCrO2, the magnetostriction associated with the formation of the single-q order was reported to be dL/L~10^-4 (J. Phys. Soc. Jpn. 78, 113710 (2009)). The authors should provide the resolution limit of the neutron powder diffraction measurements and lower limit of the detectable lattice distortion. Since the formation of the double-q order relies on the assumption that there is no lattice distortion, the proposed picture of the magnetic structure is not convincing enough.
> Normally, to confirm the formation of the multi-q states, measurements of higher-order magnetic reflections indexed by q1+q2 (=(q,0,0)+(0,q,0)=(q,q,0)) are carried out (for instance Nat. Nanotech. 15, 444 (2020).

Response: Thank you for suggesting to look for higher-order magnetic reflections, which we previously did not check for. The strong magnetic superstructure reflection (110)-(q00) at d = 3.27 Å is found to have an associated higher-order (110)-(qq0) reflection at a d-spacing of 5.79 Å, where a small step in magnetic intensity is visible in the neutron diffraction difference data. We marked both reflections in Figure 3a. We added an additional discussion the neutron diffraction section. We note that single crystal diffraction is required to show that the higher order magnetic reflection is at the correct position in 3D Q-space; nitrides, however, are often microcrystalline from high-pressure synthesis. Additionally, we collected high-resolution synchrotron diffraction data to investigate any lattice distortion; the structure remains tetragonal down to 4 K determined with an accuracy in the order of 10^{-5} Å, which should reveal even slight metric distortions. Both, the neutron diffraction and the synchrotron diffraction, give evidence that the double k magnetic structure is likely favoured over a single-k structure. We added the discussion to the main text as well as Supplementary Information.

> (4) It should also be pointed out that the sinusoidal magnetic structures contain the magnetic sites whose magnetic moments are disordered, regardless of the number of q-vectors. The authors ascribed the upturn of the magnetic susceptibility below TN to the partially paramagnetic moments. This kind of partial-orders often appear in frustrated magnetic systems at finite temperatures. As the temperature is decreased the fluctuating magnetic moments tend to be frozen, and the magnetic modulation would be squared up, which often results in higher harmonic peaks indexed as 2q, 3q… Although this kind of higher harmonic peaks are expected to be weak, the authors should mention whether they were observed or not.

Response: Thank you for pointing this out. We have not observed higher harmonic peaks and now mention this in the main text.

Point-by-Point Response Letter

Manuscript Title: Complex magnetism in a transition metal nitride
Manuscript ID: NCOMMS-26-032407A

Reviewer #1 (Remarks to the Author):

I have reviewed the revised manuscript. I find that the authors largely addressed my concerns from the previous report, thus I recommend publication assuming that the authors has answered the concerns from the other referees in a satisfactory manner. However, I do not want mention that some of the authors' discussion of the DFT calculations seems a bit unclear and of questionable validity. For example, in the reply letter they say "a proper description of the respective experimentally observed magnetic moments requests to treat the functional of each cobalt atom independently. As correlation and exchange cannot be properly described by the available sorts of functionals, an individual Hubbard U parameter must be included for every cobalt site. Accordingly, such a procedure also means that a Hubbard U screening has to be accomplished for each cobalt site within the supercell. Unfortunately, such computations represent a problem to the architecture of the used VASP code."

I don't think that one can treat the "functional of each cobalt atom independently" within a standard DFT code, to my knowledge. One would choose a functional (e.g. PBE or LDA) and use that choice for all elements in the calculation. I'm guessing that the authors are saying that they are using DFT+U to account for the on-site correlation on Co, which is correct. However, this is a fairly standardized approach, where one sets U on each magnetic site (in this case Co), so it is not clear to me why this would pose a problem "to the architecture of the vasp code." Since the primary novelty of the paper is based on the experimental results, I don't think these issues should hinder the paper from moving forward. On the other hand, it would be useful if the authors could clarify what they mean by their statements regarding DFT and its limitations for their material, in order to improve confidence that the calculations were conducted in the appropriate way.

Response: We thank the reviewer for this comment and would like to clarify our wording. We were not referring to the choice of different exchange-correlation functionals, but to the DFT+U approach with an individual Hubbard U parameter for each cobalt site.
Also, we fully agree that the DFT+U approach is standard practice for simple systems such as NiO. However, a thorough Hubbard U screening for every site is requested in order to properly solve the self-interaction error that turns out to become a severe problem of DFT-based computations, particulary, in the case of magnetically complex scenarios. In the case of our $7\times7\times1$ superstructure, there are 98 crystallographically independent Co sites to be treated, requiring 98 individual and independent U screenings. This scale was the actual practical obstacle: test calculations failed to converge even after several weeks of runtime. We hope this clarifies our original statement, and we thank the reviewer for prompting this clarification.

Reviewer #2 (Remarks to the Author):

I co-reviewed this manuscript with one of the reviewers who provided the listed reports. This is part of the Nature Communications initiative to facilitate training in peer review and to provide appropriate recognition for Early Career Researchers who co-review manuscripts.

Response: We thank you for reviewing our manuscript.

Reviewer #3 (Remarks to the Author):

The authors have improved the manuscript and clarified the points raised in my previous report. They have also provided additional support for their main claim regarding the possibility of a double-k magnetic structure. In my view, either a single-k or a double-k magnetic state would make this system interesting, and presenting the details of both models provides a clear assessment of the possible interpretations consistent with the available data. Considering the novelty of Ca2CoN2 and its unusual incommensurate magnetic state, I believe that the manuscript is of sufficient interest and significance and is now in good shape for publication.

Response: We thank you for reviewing our manuscript.

Reviewer #4 (Remarks to the Author):

The authors have adequately addressed the issues raised in the previous review round, and revised the manuscript accordingly. In particular, the additional high-resolution XRD measurements using synchrotron radiations source and the assignment of the (q,q,0) reflection are very valuable. The double-q magnetic structure is now plausible, and the discovery of the multi-q magnetic order in nitromatallates would attract attention in this research field. The revised manuscript would deserve to be published in Nat. Commun.

Response: We thank you for reviewing our manuscript.
